# Metabolome and Transcriptome Analysis Reveals the Regulatory Effect of Magnesium Treatment on EGCG Biosynthesis in Tea Shoots (*Camellia sinensis*)

**DOI:** 10.3390/plants14050684

**Published:** 2025-02-23

**Authors:** Zixuan Feng, Zhuan Li, Rui Yan, Nan Yang, Meichen Liu, Yueting Bai, Yuyuan Mao, Chengzhe Zhou, Yuqiong Guo, Yulin Zeng, Yuhang Ji, Yangshun Lin, Jiayong Chen, Shuilian Gao

**Affiliations:** 1 Anxi College of Tea Science & College of Horticulture, Fujian Agriculture and Forestry University, Fuzhou 350002, China; 18650318466@163.com (Z.F.); i0217z@163.com (Z.L.); yr872318@163.com (R.Y.); 18065359317@163.com (N.Y.); 13792894565@139.com (M.L.); 18548584590@163.com (Y.B.); m_yuyuan@163.com (Y.M.); chengzhechou@fafu.edu.cn (C.Z.); guoyq828@163.com (Y.G.); 18805985391@163.com (Y.Z.); jiyuhang010811@163.com (Y.J.); 2 Fujian Collaborative Innovation Center for Green Cultivation and Processing of Tea Tree in Colleges and Universities, Quanzhou 362406, China; 3 Quanzhou Special Talent Innovation Laboratory of Fujian Richun Industrial Co., Ltd., Quanzhou 362000, China; chenjianchun@rctea.com; 4 Anxi County Tea Industry Development Center, Quanzhou 362300, China; 13506939977@163.com

**Keywords:** tea (*Camellia sinensis* (L.) Kuntze), EGCG biosynthesis, multi-omics, WGCNA, transcription factors

## Abstract

Epigallocatechin-3-O-gallate (EGCG) is an important ingredient that indicates tea quality and has healthcare functions. Magnesium nutrition can improve the quality and yield of tea plants, but its regulatory role in the biosynthesis of EGCG in tea plants has not been clarified. Herein, we performed a comprehensive analysis of the metabolomics and transcriptomics of the shoots of ‘Huangdan’ at five magnesium concentrations: L1-L5 (0, 0.15, 0.45, 0.6, and 0.9 mmol/L mg^2+^, respectively). The results showed that the EGCG content of tea shoots treated with low magnesium concentrations was higher compared to those treated with high magnesium concentrations. The contents of related metabolites such as p-coumaric acid and cyanide in the EGCG synthesis pathway increased in the L4 and L5 treatment groups, while those of dihydroquercetin, dinnamic acid, and epicatechin increased significantly in the L2 and L3 treatment groups. Under the influence of magnesium treatment, the biosynthesis of EGCG was affected by a series of structural genes: *CsPAL (HD.01G0005520)*, *HD.02G0024350)*, *Cs4CL (HD.15G0008250*, *HD.13G0010220)*, *CsDFR (HD.04G0026220)*, *CsANS(HD.12G0016700)* with *CsaroDE (HD.03G0002480)*-positive regulation, and *CsPAL (HD.13G0009900*, *HD.06G0008610)*, *CsC4H (HD.06G0017130)*, *Cs4CL (HD.02G0027390*, *HD.04G0003270)*, *CsCHS (HD.10G0022640)*, *CsCHI (HD.01G0011100)*, *CsF3′H (HD.15G0015490)*, *CsF3′5′H (HD.13G0004300)*, *CsANS (HD.07G0023630)*, and *Csaro B (HD.01G0028400)* with *CsSCPL (HD.01G0041070)*-negative regulation. Transcription factors *MYB 44* and *WRKY 17* may play a key role in EGCG biosynthesis, which is significantly induced by magnesium nutrition in tea tree shoots. This study elucidates the effect of magnesium nutrition on EGCG biosynthesis in tea plants and provides key candidate transcription factors to provide a reference for further research on high-EGCG tea varieties to improve tea quality.

## 1. Introduction

Nutrients play an irreplaceable role in the growth of tea plants [*Camellia sinensis* (L.) Kuntz] and are closely related to the quality and yield of tea. Tea is the second most popular beverage in the world after water, with lipid-lowering, weight loss, anti-inflammatory, antibacterial, antioxidant, and other effects [1], and its quality affects human health [2]. Catechins are polyphenolic compounds, accounting for 12–14% of the dry weight of tea leaves. Epigallocatechin gallate (EGCG) is the most abundant catechin and is a key factor in the sensory and health qualities of tea [3]. It has anticancer, antibacterial, and antihypertensive effects [4,5]. The biosynthesis of EGCG is based on a galacyl transacylation reaction at position 3 on the C ring of non-ester catechins, which involves a two-step reaction. First, glycosyltransferase (*UGGT*) catalyzes the formation of galloacylglucose (β-glucogallin) from gallic acid and glucose diphosphate urate, followed by 1-*O*-galoyl-β-d-glucose *O*-galactosyltransferase catalyzing the reaction of non-ester catechins with galloylglucose to synthesize ester catechin EGCG [6,7].

The accumulation of EGCG is affected by multiple factors such as nutrient supply, environmental factors, and stress responses. When the concentration of sodium selenite (Na_2_SeO_3_) exceed 0.125 mg·kg^−1^, the selenium content of tea increased first and then decreased with the increase of Na_2_SeO_3_ use, and the contents of amino acids, polyphenols, and soluble sugar in tea increased significantly [8]. The exogenous application of calcium enhances drought tolerance in tea plants by influencing galactitol biosynthesis pathways and mediating the regulation of the stomatal pore size of protective cells [9]. As an essential nutrient element for the growth of tea plants, magnesium has an irreplaceable role in the biosynthesis of EGCG. Li et al. confirmed that short-term magnesium deficiency increases the concentrations of polyphenols, free amino acids, and caffeine in tea, but decreases the chlorophyll content. Long-term magnesium deficiency decreases the content of the above components, but as the phenol–ammonia ratio increases, bitterness and astringency increase, and the umami taste decreases, affecting the taste of the tea [10,11]. However, the regulatory mechanism of magnesium nutrition on EGCG biosynthesis is still unclear. Therefore, it is of great significance to systematically study the response mechanism of EGCG to magnesium nutrition to improve the quality of tea.

With the wide application of multi-omics technology, a variety of nutrient-responsive genes have been identified in crops, such as transcription factor (TF) genes and hormone metabolism-related genes. In citrus fruit development, magnesium regulates the metabolism of colored layer sugars by up-regulating the expression of AI, SS, and SPS genes, and promotes the accumulation of colored layer fructose, glucose, and sucrose, thereby affecting the color of the citrus fruit exocarp [12]. Wamoto Masao’s study showed that the transcription factor RDD3 helps improve the uptake and accumulation of ammonium nitrogen and magnesium in rice and enhances its drought resistance [13]. *CitCHS* in the flavonoid pathway of sweet orange is crucial under magnesium stress, and is a backbone gene in the flavonoid biosynthesis pathway, which has a significant impact on the synthesis of flavonoids and other flavonoids [14]. Magnesium stress experiments in mulberry showed that differentially expressed genes were mainly enriched in flavonoid biosynthesis pathways, secondary metabolite biosynthesis pathways, and other metabolic pathways. Among them, the nitrate reductase gene (*MmNIA*) helps mulberry trees to cope with magnesium stress [15]. Guo Lei et al. showed that the application of magnesium can enhance the utilization rate of light energy, improve the activity of alanine ammonia–lyase (*PAL*), and promote the accumulation of catechins, thereby improving the quality of tea [16]. The above results confirmed that TFs had an effect on crop quality, yield, and resistance under magnesium nutrient treatment.

In this study, five new shoots of tea tree at different magnesium concentrations were used for combined metabolome and transcriptome analysis to screen out the key TFs associated with EGCG biosynthesis. Based on real-time quantitative PCR (qRT-PCR) technology, the role of the screened genes in the biosynthesis of EGCG from tea shoots under magnesium nutrition was confirmed. This study provides a reference for exploring the practical application of magnesium nutrition in tea plants.

## 2. Results

### 2.1. Evaluation of Main Biochemical Components in Tea Shoots Treated by Magnesium

High-performance liquid chromatography (HPLC) was used to detect the results of caffeine, gallic acid (GA), catechins, tea polyphenols, and free amino acids in tea leaves at different concentrations of Mg^2+^ (L1–L5: 0, 0.15, 0.45, 0.6, and 0.9 mmol/L, respectively). As shown in Table 1, the caffeine content decreased with an increase in Mg^2+^ concentration. EGCG content increased significantly in 0.15 mmol/L and 0.45 mmol/L treatments, and decreased gradually in 0.6 mmol/L and 0.9 mmol/L treatments. After the experimental treatment, the contents of the six identified catechins showed a gradual decreasing trend, and the content was lowest in the 0.6 mmol/L treatment. The total amount of free amino acids increased first, then decreased, and increased with the increase in magnesium concentration, and the maximum increase was 5% and 13.7% after L4 (0.6 mmol/L) and L5 (0.9 mmol/L) treatments, respectively, with significant changes (*p* < 0.05). With the increase in magnesium concentration, the content of tea polyphenols increased after L2 treatment, showing a parabolic trend of first increasing and then decreasing.

### 2.2. Evaluation of Magnesium and Chlorophyll Contents in Tea Shoots Treated by Magnesium

**Figure 1:**
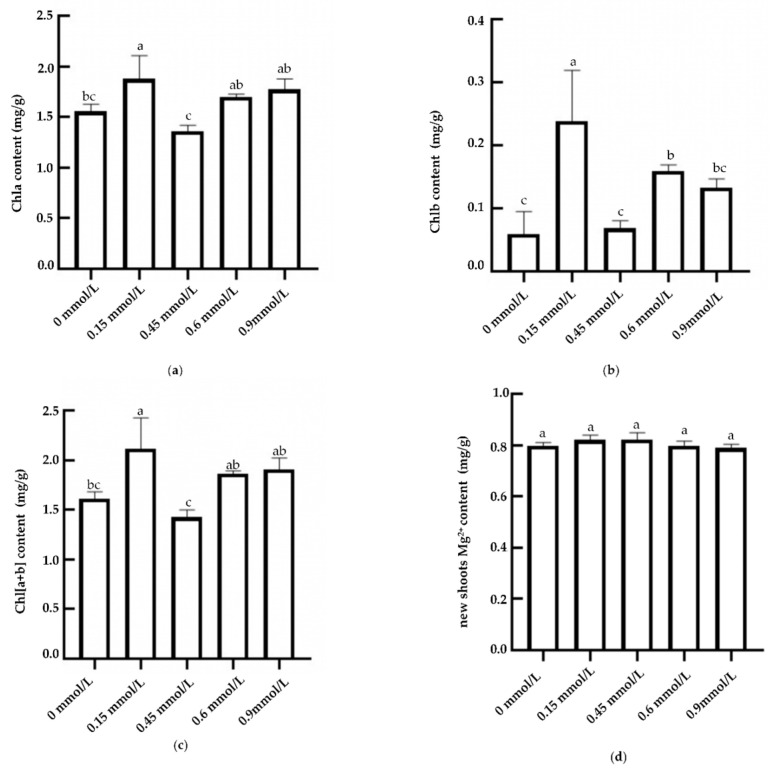
Mg^2+^ and chlorophyll contents of new shoots in tea treated by magnesium. Notes: L1–L5 (0, 0.15, 0.45, 0.6, and 0.9 mmol/L mg^2+^, respectively); different lowercase letters indicate significant differences between treatments (*p* < 0.05). (**a**) Chlorophyll a and (**b**) chlorophyll b, (**c**) total chlorophyll, and (**d**) Mg^2+^ contents in tea shoots treated by magnesium.

The results of chlorophyll measurement showed that the levels of chlorophyll a (Chla), chlorophyll b (Chlb), and total chlorophyll [Chl(a + b)] showed a relatively consistent trend under different Mg^2+^ treatment conditions. As the concentration of Mg^2+^ increased, the content of various pigments first increased, decreased, and then increased, reaching a peak at 0.15 mmol/L, which was significantly higher than that at magnesium deficiency (0 mmol/L) and higher concentrations (0.45 mmol/L) (Figure 1a–c). The levels of chlorophyll a, chlorophyll b, and total chlorophyll were significantly higher in the 0.15 mmol/L treatment than those in the 0.45 mmol/L, 0.6 mmol/L, and 0 mmol/L treatments, and there was no significant difference in the chlorophyll a content in the 0.6 mmol/L and 0.9 mmol/L treatments, but the chlorophyll a content in the 0.15 mmol/L treatment was significantly higher than that in the 0.45 mmol/L and 0 mmol/L treatments, which increased by 38.5% and 20.65%, respectively. Chlorophyll b was significantly increased in the 0.15 mmol/L treatment compared to the 0.45 mmol/L treatment.

The Mg^2+^ content in tea leaves increased first and then decreased after irrigation and application of different concentrations of magnesium nutrient solution (Figure 1d). The Mg^2+^ content in tea leaves increased rapidly to 0.82 mg/kg in the 0.15 mmol/L treatment and reached the same concentration (0.82 mg/kg) in the 0.45 mmol/L treatment. With the increase in magnesium nutrient concentration (0.6~0.9 mmol/L), the Mg^2+^ content in leaves began to decrease gradually, and the Mg^2+^ content decreased to 0.78 mg/kg in the 0.9 mmol/L treatment.

### 2.3. Analysis of Widely Target Metabolomic in Tea Shoots Treated by Magnesium

**Figure 2:**
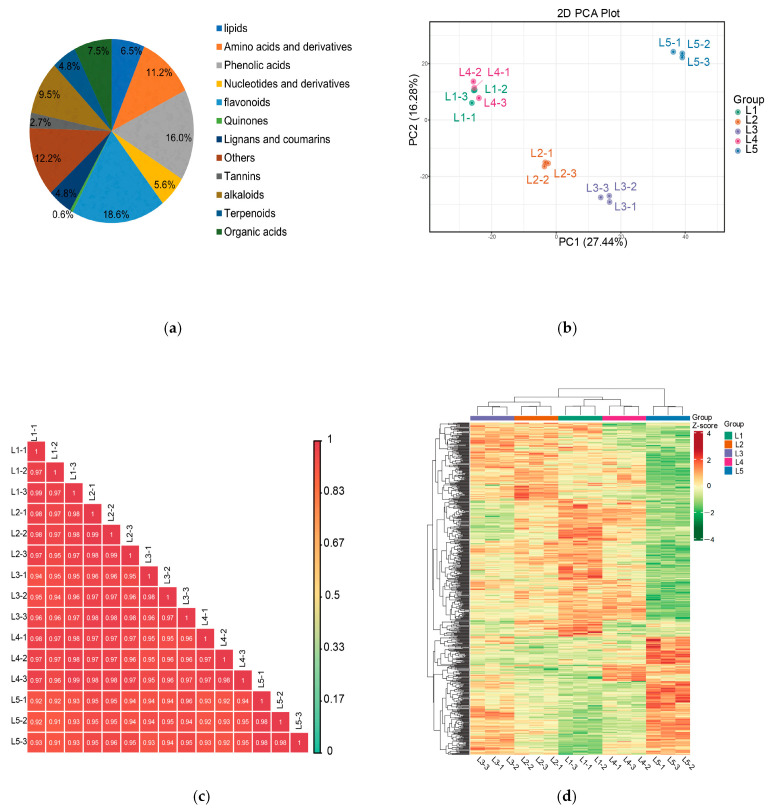
Analysis of widely targeted metabolomics from tea shoots treated by magnesium. Notes: L1–L5 (0, 0.15, 0.45, 0.6, and 0.9 mmol/L mg^2+^, respectively), same below. (**a**) Different types and proportions of pie charts of all non-volatile metabolites in tea shoots treated by magnesium. (**b**) Principal component analysis of all samples in tea shoots treated by magnesium (the x- and y-axes represent the first and second principal components, respectively). (**c**) Correlation analysis from 15 samples in tea shoots treated by magnesium. (**d**) Cluster heat maps of all non-volatile metabolites in tea shoots treated by magnesium.

A total of 1249 metabolites in 12 categories were identified by the metabolite analysis of samples treated with different magnesium concentrations (L1, L2, L3, L4, and L5) using the UPLC-Q-TOF/MS method (Figure 2a). These metabolites included 232 flavonoids, 200 phenolic acids, 152 other substances, 118 alkaloids, 81 lipids, 140 amino acids and their derivatives, 61 terpenoids, 61 lignans and coumarins, 93 organic acids, 70 nucleotides and their derivatives, 34 tannins, and 7 quinones. Flavonoids made up 18.6% of the metabolites. Magnesium concentration had a strong effect on flavonoids. We performed PCA for all samples and found that PC1 and PC2 explained 43.72% of the total variance of the differential metabolites (Figure 2b). L2, L3, and L5 were well separated, while L1 and L4 were close, indicating that the metabolite profiles of new shoots in the Mg-treated tea tree differed mainly in L2, L3, and L5. The PCA results reflected the metabolite differences among the five groups. Pearson’s analysis showed the high reproducibility of the samples (Figure 2c). Hierarchical clustering analysis revealed significant differences in non-volatile metabolites between different magnesium treatments in the heatmap (Figure 2d), in which L2, L3, and L5 were clearly separated, and L1 and L4 were clustered together, which was consistent with the PCA results.

### 2.4. Analysis of Non-Volatile Metabolites from Differential Accumulation in Tea Shoots Treated by Magnesium

**Figure 3:**
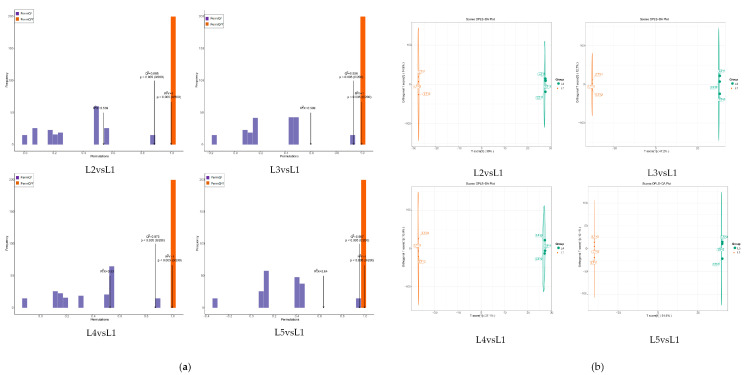
Orthogonal partial least-squares discriminant analysis from tea shoots treated by magnesium. Notes: (**a**) Test of fitting degree of tea metabolite OPLS-DA model in tea shoots treated by magnesium. (**b**) Analysis of metabolites in tea by OPLS-DA model in tea shoots treated by magnesium.

The orthogonal partial least-squares discriminant analysis (OPLS-DA) model (Figure 3a) was used to screen out the key compounds with the most significant changes after different magnesium concentrations. The results showed that the R^2^Y and Q^2^ values of the four models (L2 vs. L1, L3 vs. L1, L4 vs. L1, and L5 vs. L1) reached significant levels, and the models had a good fit and high confidence, which could be used for further analysis. To further understand the differences between the five groups of samples, OPLS-DA was performed and the evaluation plot results (Figure 3b) showed that it effectively separated samples L1, L2, L3, L4, and L5 into four different regions, while L1 samples were in different regions. It can be seen that there are significant differences in metabolites between L1, L2, L3, L4, and L5.

**Figure 4:**
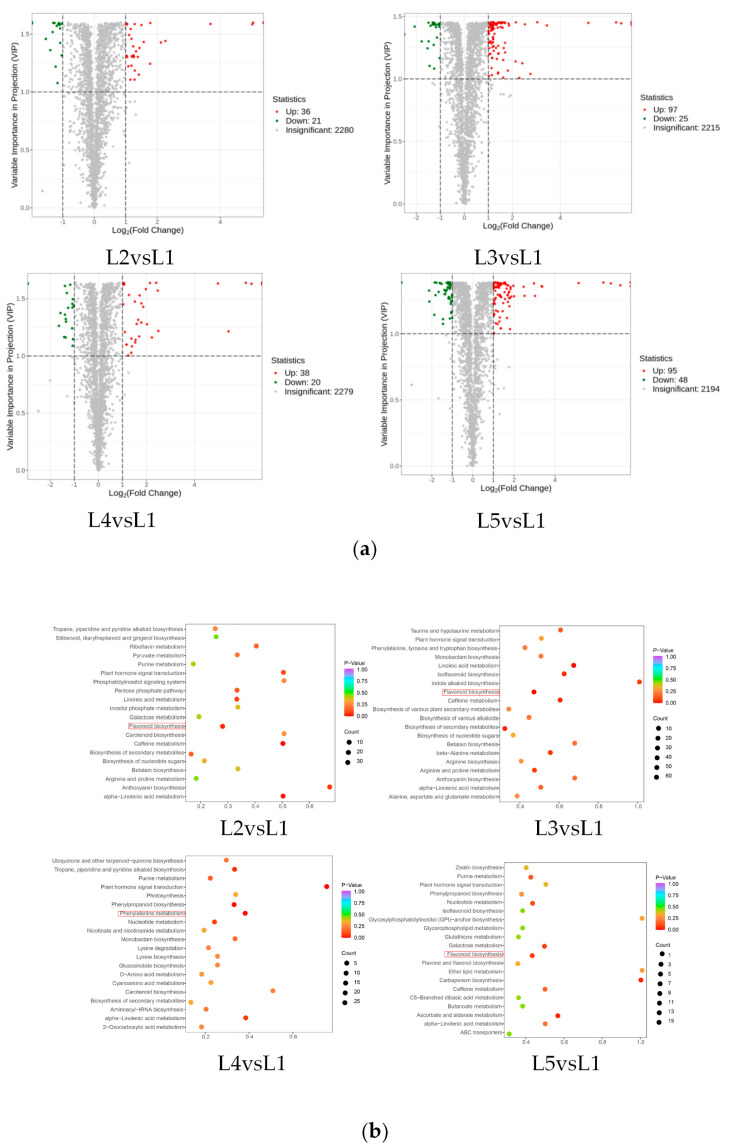
SCMs analysis for different comparison groups (L2 vs. L1, L3 vs. L1, L4 vs. L1, and L5 vs. L1) from tea shoots treated by magnesium. Notes: (**a**) Volcanic maps and (**b**) KEGG enrichment analysis of SCMs (L2 vs. L1, L3 vs. L1, L4 vs. L1, and L5 vs. L1) in tea shoots treated by magnesium. Red box represents the main enrichment path.

In order to visually show the significantly changed metabolites (SCMs) between the five groups of tea samples, a volcano map was drawn for the SCMs between the five groups of tea samples (Figure 4a), and a total of 380 SCMs were identified in the four comparison groups, which could be divided into lipids, organic acids, terpenoids, alkaloids, tannins, others, lignans and coumarins, flavonoids, nucleotides and their derivatives, phenolic acids, and amino acids and their derivatives. A total of 57 SCMs were screened for L2 vs. L1 (36 up-regulated and 21 down-regulated), 122 SCMs (97 up-regulated and 25 down-regulated) were screened for L3 vs. L1, 58 SCMs were screened for L4 vs. L1 (38 up-regulated and 20 down-regulated), and 143 SCMs (95 up-regulated and 48 down-regulated) were screened for L5 vs. L1. KEGG pathway enrichment analysis was performed on the SCMs of the four comparison groups (L2 vs. L1, L3 vs. L1, L4 vs. L1, and L5 vs. L1) according to the *p* value (Figure 4b). They were mainly distributed in 20 metabolic pathways, and the SCMs of the comparison groups were mainly enriched in the pathways of flavonoid biosynthesis and phenylpropane biosynthesis.

### 2.5. Identification and Analysis of Key Differential Metabolites in Tea Shoots Treated by Magnesium

In order to identify the key differential metabolites in the four comparison groups, the fold changes in the metabolites in the four comparison groups were compared. A total of 9 significantly differential metabolites, including flavonoids, phenolic acids, organic acids, terpenes, alkaloids, lipids, and other compounds, were identified in the four comparison groups, among which flavonoid compounds were the main differential metabolites (Supplementary Tables S1–S4).

**Figure 5:**
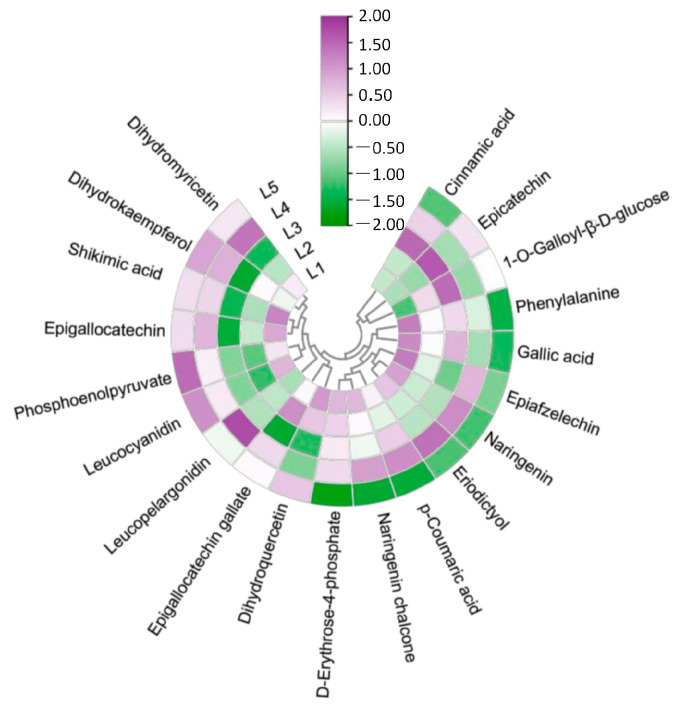
SCMs of the EGCG synthesis pathway in tea shoots treated by magnesium. Notes: The color bar represents the normalized fold change values.

In order to explore the regulatory mechanism of magnesium treatment on EGCG synthesis, the metabolites related to EGCG synthesis were screened and visualized (Figure 5). These differential metabolites included phenylalanine, cinnamic acid, p-coumaric acid, naringenin chalcone, naringenin, aromadendrin, leucopelargonidin, epiafzelechin, eriodictyol, dihydroquercetin, leucocyanidin, epicatechin, dihydromyricetin, epigallocatechin, epigallocatechin gallate, shikimic acid, phosphoenolpyruvate, d-erythrose-4-phosphate, gallic acid, and 1-*O*-galloyl-β-d-glucose. Cinnamic acid, epicatechin, 1-*O*-galloyl-β-d-glucose, naringenin, eriodictyol, p-coumaric acid, naringenin chalcone, epigallocatechin gallate, and d-erythrose-4-phosphate first increased and then decreased with the increase in magnesium concentration. Some substances such as dihydroquercetin, phosphoenolpyruvate, leucocyanidin, and leucopelargonidin showed a decreasing trend. Among them, the contents of eriodictyol, p-coumaric acid, phosphoenolpyruvate, and leucocyanidin increased in the L4 and L5 treatment groups, and the contents of dihydroquercetin, cinnamic acid, and epicatechin increased significantly in the L2 and L3 treatment groups.

### 2.6. Transcriptomic Analysis in Tea Shoots Treated by Magnesium

High-throughput RNA-seq was conducted on five one-bud two-leaf RNA samples (L1–L5), each with three biological replicates. In the transcriptome analysis, the range of raw read segments was from 72,025,138 to 55,709,340. The clean reads varied between 47,970,904 and 71,269,586, while the clean bases ranged from 7,085,001,258 to 10,380,500,774. The GC content varied from 45.3% to 46.64%. All Q30 values exceeded 95%, indicating that the experimental results are highly reliable and suitable for further experimental analysis.

**Figure 6:**
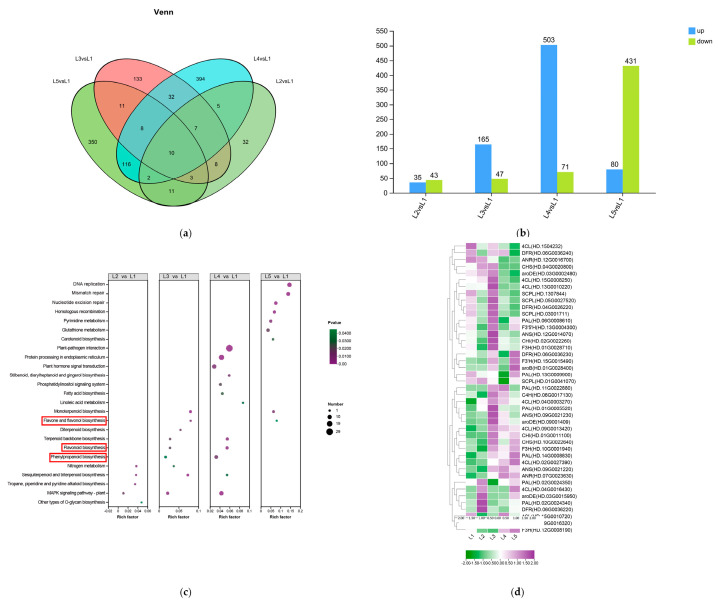
Analysis of differentially expressed genes (DEGs) and functional annotation in tea shoots treated by magnesium. Notes: L1–L5 (0, 0.15, 0.45, 0.6 and 0.9 mmol/L mg^2+^), same below; (**a**) Venn diagram of DEGs from different comparison groups (L2 vs. L1, L3 vs. L1, L4 vs. L1, and L5 vs. L1) in tea shoots treated by magnesium. (**b**) Histogram of the number of up-regulated and down-regulated DEGs in different comparison groups (L2 vs. L1, L3 vs. L1, L4 vs. L1, and L5 vs. L1) in tea shoots treated by magnesium. Among them, blue represents the up-regulated DEGs, and green represents the down-regulated DEGs. (**c**) KEGG pathway enrichment for DEGs from different comparison groups (L2 vs. L1, L3 vs. L1, L4 vs. L1, and L5 vs. L1) in tea shoots treated by magnesium. (**d**) Cluster heat map of DEGs related to EGCG biosynthesis in tea shoots treated by magnesium. Red boxs represents the main enrichment path.

The Venn diagram (Figure 6a) demonstrated that four comparison groups (L2 vs. L1, L3 vs. L1, L4 vs. L1, and L5 vs. L1) were 31, 392, 137, and 343 differentially expressed genes (DEGs) responding to different concentrations of magnesium treatment. Compared with L1 (0 mmol/L), there were 35 (43), 165 (47), 503 (71), and 80 (431) up-regulated (down-regulated) DEGs for L2 (0.15 mmol/L), L3 (0.45 mmol/L), L4 (0.6 mmol/L), and L5 (0.9 mmol/L) all had a large number of DEGs. The treatments L3 (0.45 mmol/L), L4 (0.6 mmol/L), and L5 (0.9 mmol/L) all had a large number of DEGs. More up-regulated DEGs than down-regulated DEGs were found in the L3 (0.45 mmol/L) and L4 (0.6 mmol/L) treatments. However, in the presence of L2 (0.15 mmol/L) and L5 (0.9 mmol/L) treatments, there were fewer up-regulated DEGs (Figure 6b).

The KEGG functional enrichment analysis revealed enrichment in the front 25 metabolic pathways (Figure 6c). The results showed that compared with the L1 treatment group, DEGs were mainly enriched in flavonoids, flavonols biosynthesis, and phenylpropanoid biosynthesis pathways. We examined the expression of genes involved in the phenylalanine, flavonoid, and shikimic acid pathways. The expression pattern of the corresponding genes is shown in Figure 6d. In the EGCG synthesis pathway, 19 DEGs were involved in the phenylpropanoid biosynthesis pathway. Most differential genes were first increased and then decreased with increasing magnesium intake. Among them, *CsC4H (HD.06G0017130)* and *CsPAL (HD.01G0005520)* were significantly up-regulated after L3 treatment. A total of 20 DEGs were involved in the flavonoid pathway. Among them, *CsaroDE (HD.03G0015950)* and *CsDFR (HD.06G0036220)* were up-regulated in L2 to promote EGCG biosynthesis. *CsDFR (HD.04G0026220)*, *CsF3′5′H (HD.13G0004300)*, *CsANS (HD.12G0014070)*, *CsCHI (HD.02G0022260)*, *CsF3H (HD.01G0028710)*, *CsANS (HD.09G0021230)*, and *CsaroDE (HD.09001409)* were up-regulated in L3 treatment to inhibit EGCG content. Interestingly, *CsCHS (HD.10G0022640)* and *CsF3′H (HD.15G0015490)* were significantly down-regulated after L3 treatment. *CsDFR (HD.06G0036230)*, *CsF3′H (HD.15G0015490)*, and *CsaroB (HD.01G0028400)* were up-regulated after L5 treatment.

### 2.7. Correlation Analysis of Related Metabolites and Their Synthesis-Related Gene Expressions in Tea Shoots Treated by Magnesium

**Figure 7:**
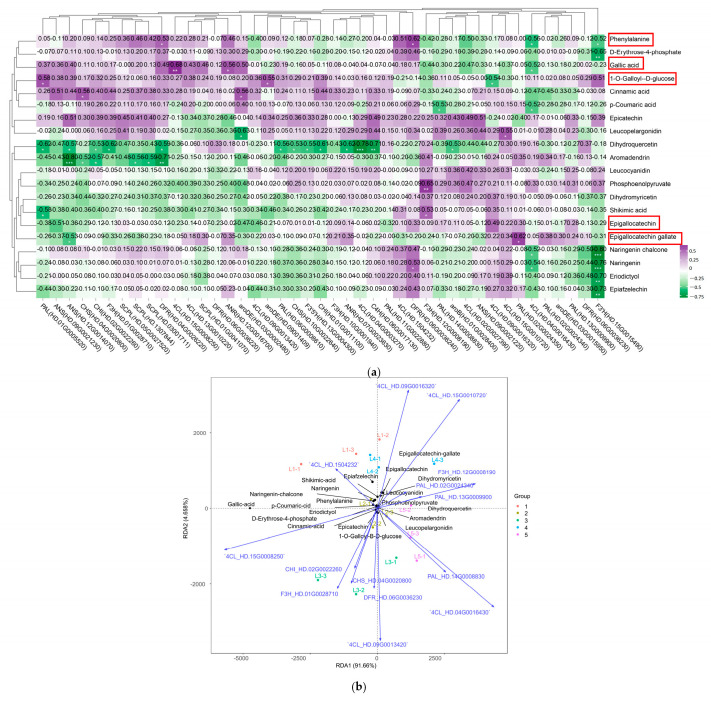
Screening of the main contributing genes for EGCG biosynthesis in tea shoots treated by magnesium. (**a**) Intergroup correlation analysis of 20 flavonoids and 43 flavonoid synthesis-related genes in tea shoots treated by magnesium. (**b**) Redundancy analysis (RDA) of 20 flavonoids and related synthetic genes from 15 samples in tea shoots treated by magnesium. * means significant difference, * *p* < 0.1, ** *p* < 0.05 and *** *p* < 0.01. Red boxes represents metabolites that change significantly in the EGCG synthesis pathway.

Through the combined analysis of transcriptomics and metabolomics, it was found that the EGCG biosynthesis pathway was associated with a total of 43 structural genes (Supplementary Table S5) and 20 flavanol compounds (Figure 5). A correlation analysis was performed between the expression of 43 genes and the content of 20 flavanol compounds (Figure 7a). The results indicated that *CsDFR (HD.04G0026220*, *HD.06G0036240)* had a significant positive correlation with phenylalanine. Similarly, *Cs4CL (HD.15G0008250)* and *CsANR (HD.12G0016700)* were significantly positively correlated with gallic acid. On the other hand, *Cs4CL (HD.09G0016320)* showed a significant negative correlation with 1-*O*-galloyl-β-d-glucose. Furthermore, *CsPAL (HD.02G0024350)* was significantly positively correlated with epigallocatechin gallate, while *CsANS (HD.09G0021230)* was negatively correlated with epigallocatechin.

A redundancy analysis (RDA) was conducted to analyze the relationship between EGCG synthesis pathway-related genes and metabolites, which was used to down-regulate 43 genes of flavanols and synthesis pathway species (Figure 7b). The results revealed that 14 genes, namely *Cs4CL (HD.09G0016320)*, *Cs4CL (HD.15G0010720)*, *Cs4CL (HD.1504232)*, *CsPAL (HD.02G0025340)*, *CsF3H (HD.12G0008190)*, *CsPAL (HD.13G0009900)*, *Cs4CL (HD.15G0008250)*, *CsCHI (HD.02G0022260)*, *CsF3H (HD.01G0028710)*, *CsCHS (HD.04G0020800)*, *CsDFR (HD.06G0036230)*, *Cs4CL (HD.09G0013420)*, *CsPAL (HD.14G0008830)*, and *Cs4CL (HD.04G0016430)*, play significant roles in EGCG biosynthesis.

The RDA results demonstrated that the independent variable matrix (genes) explained 96.318% (RDA1 + RDA2) of the dependent variable matrix (flavanol compounds), indicating a high reliability of the analyzed results. The findings revealed that 14 key genes involved in EGCG biosynthesis were correlated with 20 flavanol compounds. The genes were divided into two categories based on their spatial distribution. The first category includes *Cs4CL (HD.1504232)*, *Cs4CL (HD.09G0016320)*, *Cs4CL (HD.15G0010720)*, *CsF3H (HD.12G0008190)*, *CsPAL (HD.13G0009900)*, and *CsPAL (HD.02G0025340)*. The second category comprises *Cs4CL (HD.15G0008250)*, *CsCHI (HD.02G0022260)*, *CsF3H (HD.01G0028710)*, *CsCHS (HD.04G0020800)*, *CsDFR (HD.06G0036230)*, *Cs4CL (HD.09G0013420)*, *CsPAL (HD.14G0008830)*, and *Cs4CL (HD.04G0016430)*. There was a significant positive correlation between genes within the same group, and a significant negative correlation between genes in different groups. In L1, the expression level of *Cs4CL (HD.1504232)* was significantly correlated with epiafzelechin, shikimic acid, p-coumaric acid, naringenin chalcone, naringenin, phenylalanine, and eriodictyol. In L3, the expression levels of *Cs4CL (HD.15G0008250)*, *CsCHI (HD.02G0022260)*, *CsF3H (HD.01G0028710)*, *CsCHS (HD.04G0020800)*, and *CsDFR (HD.06G0036230)* were significantly correlated with the levels of gallic acid, d-erythrose-4-phosphate, 1-*O*-galloyl-β-d-glucose, and cinnamic acid. In L2, L4, and L5, the expression levels of *Cs4CL (HD.09G0016320)*, *Cs4CL (HD.15G0010720)*, *CsF3H (HD.12G0008190)*, *CsPAL (HD.13G0009900)*, *CsPAL (HD.02G0025340)*, *Cs4CL (HD.09G0013420)*, *CsPAL (HD.14G0008830)*, and *Cs4CL (HD.04G0016430)* were significantly correlated with EGC, EGCG, dihydroquercetin, dihydromyricetin, leucocyanidin, phosphoenolpyruvate, aromadendrin, EC, and leucopelargonidin. The results showed that different concentrations of magnesium treatments had a significant effect on the flavanol content of new tea tree shoots.

### 2.8. Pathways of EGCG Biosynthesis in Tea Shoots Treated by Magnesium

**Figure 8:**
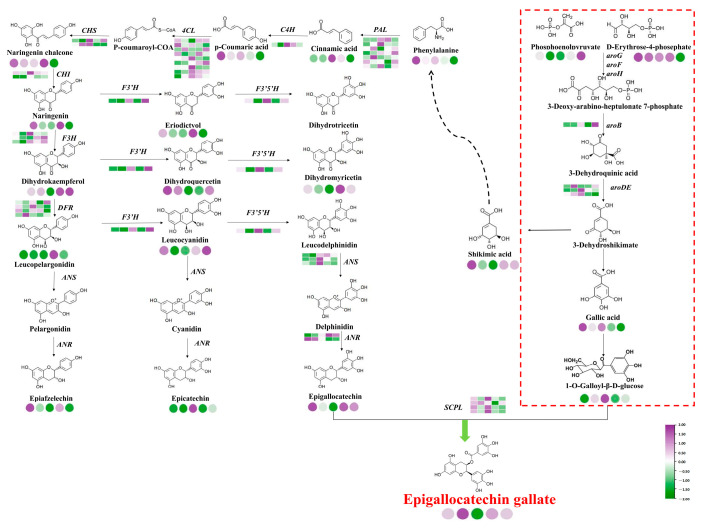
Path diagram of the regulation of EGCG biosynthesis corresponding to magnesium concentration. Notes: Circles represent metabolites, squares represent genes, purple represents up-regulation, and green represents down-regulation.

Based on the correlation analysis of related metabolites and their synthesis-related gene expression, a path for magnesium to regulate EGCG accumulation was constructed (Figure 8). Among them, 19 structural genes (7 *CsPALs*, 1 *CsC4H*, 9 *Cs4CLs*, and 2 *CsCHSs*) were involved in the phenylpropanoid pathway; 20 structural genes (2 *CsCHIs*, 3 *CsFHs*, 4 *CsDFRs*, 1 *CsF*3′*H*, 1 *CsF*3′5′*H*, 3 *CsANS*, 2 *CsANRs*, and 4 *CsSCPLs*) were involved in the flavonoid pathway, 4 structural genes (1 *CsaroB*, 3 *CsaroDEs*) were involved in the shikimic acid pathway, and most of the differentially genes were up-regulated and then down-regulated with the increase in magnesium nutrition. Among them, the expression levels of *CsC4H*, *Cs4CL*, *CsCHS*, *CsF*3′5′*H*, *CsANS*, and *CsSCPL* under L3 (0.45 mmol/L) treatment were significantly higher than those of other treatments, which promoted the accumulation of EGCG. The expression levels of *CsPAL* and *CsF*3′*H* under L5 treatment were significantly higher than those under other treatments, which inhibited the biosynthesis of EGCG.

### 2.9. Identification of Transcription Factors in Response to Magnesium Treatment

**Figure 9:**
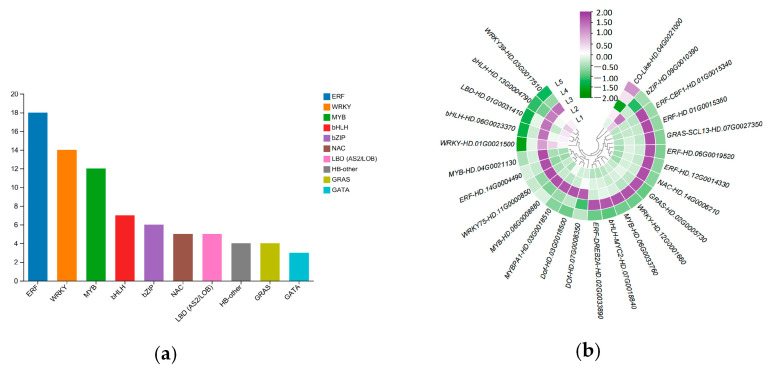
Identification and analysis of transcription factors in response to magnesium treatment. (**a**) Top 10 transcription factor family expressions in response to magnesium treatment. (**b**) TFs that may play a role in EGCG biosynthesis in tea shoots treated by magnesium.

A total of 3048 differentially expressed transcription factors were identified in five tea groups, divided into 48 categories, and the top ten transcription factor families were mainly *MYB*, *ERF*, *bHLH*, *MYB-related*, *HB-other*, *NAC*, and *WRKY* (Figure 9a). Of these, there are 25 TFs that may play roles in EGCG biosynthesis (Figure 9b), including 6 *ERFs*, 4 *WRKYs*, 4 *MYBs*, 3 *bHLHs*, 2 *GRASs*, 2 *Dofs*, 1 *NAC*, 1 *LBD*, 1 *CO-like*, and 1 *bZIP*.

### 2.10. WGCNA and Hub Gene Identification

**Figure 10:**
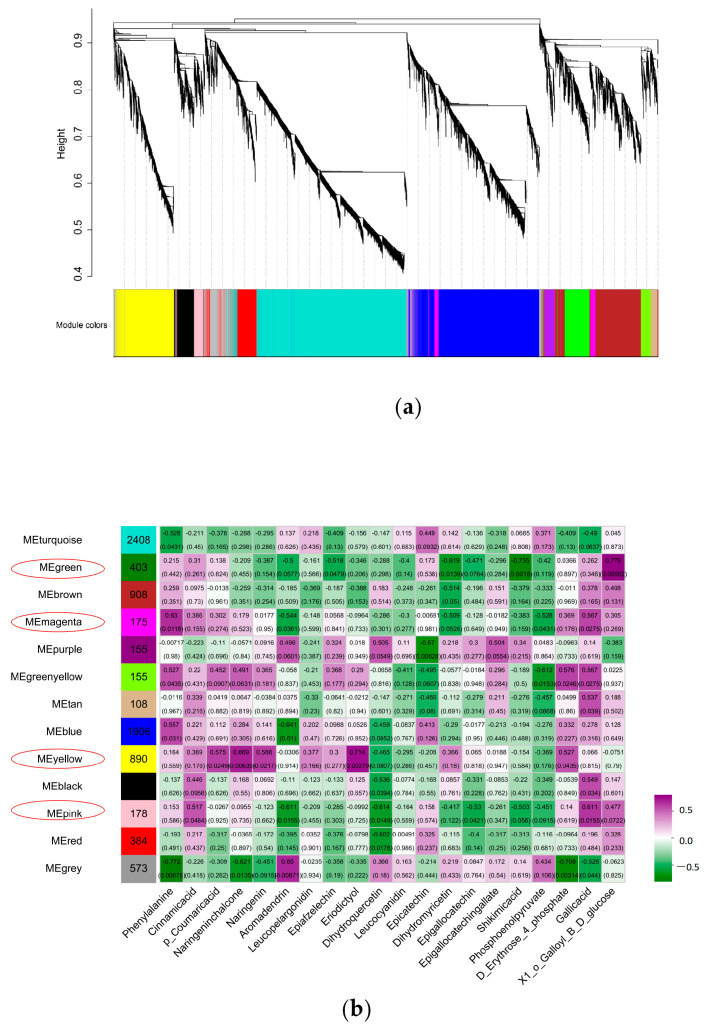

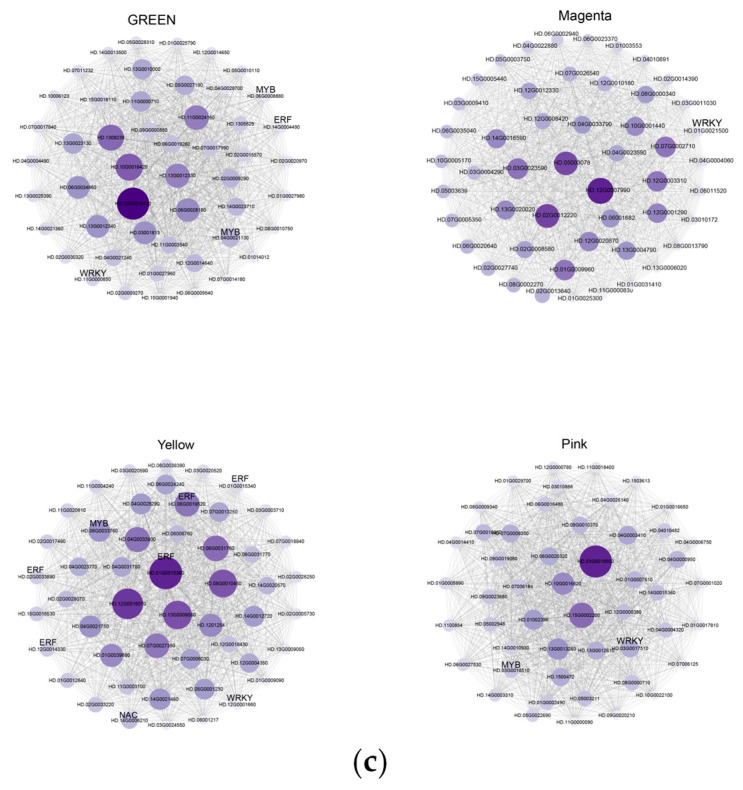
Co-expression network of SCMs and DEGs for EGCG biosynthesis in tea shoots treated by magnesium. Notes: (**a**) Construction of WGCNA co-expression network in tea shoots treated by magnesium. (**b**) Correlation analysis between modules and phenotypes (the sample clustering tree; matrix of module–metabolite associations; the abscissa represents phenotypes; the ordinate represents modules; the number of genes in the module is shown in the left box; correlation coefficients and *p*-values between modules and metabolites are shown at the row–column intersection; red means higher correlation and blue means lower correlation). (**c**) Co-expression gene clustering. (When the correlation coefficient (r) is greater than 0.8, it is believed that there is a regulatory relationship between the candidate genes and TFs. The line colors between nodes indicate the strength of the correlation).

After screening the genes with small expression fluctuations, a total of 8547 genes were classified by WGCNA into 13 co-expression gene modules, which were defined as turquoise, gray, green, brown, magenta, purple, green-yellow, tan, blue, yellow, black, pink, and red, with module sizes ranging from 108 to 2408 (Figure 10a). Through visual analysis of the correlation between 13 modules and 20 metabolites, turquoise module genes had the largest number (2408) and tan module genes had the smallest number (108); the green module was significantly correlated with 1-*O*-Galloyll-β-d-glucose. Yellow was associated with 10 flavanol metabolites, among which 2 flavanol compounds had the highest correlation (r = 0.6, *p* < 0.05). The magenta module was positively correlated with phenylalanine (r = 0.63, *p* < 0.05). The pink and gray modules were significantly positively correlated with gallic acid and aromadendrin, respectively, and the yellow module was significantly correlated with naringenin chalcone and eriodictyol (Figure 10b). Genes with high connectivity were defined as hub genes, and the top 50 genes with the highest connectivity among the four modules were selected according to the characteristic gene connectivity (KME) values to construct a co-expression network, which was visualized by Cytoscape 3.10 software (Figure 10c), and TFs were selected as the key hub genes. A total of four TFs were identified in the green module, namely two *MYB (HD.06G0008880*, *HD.04G0021130*), one *ERF (HD.14G0004490)*, and one *WRKY (HD.11G0000850)*. One *WRKY* TF *(HD.01G0021500)* was identified in the magenta module. A total of eight TFs were identified in the yellow module, including one *NAC (HD.14G0006210)*, one *MYB (HD.06G0033760)*, one *WRKY (HD.12G0001660)* and five *ERFs (HD.01G0015340*, *HD.01G0015360*, *HD.02G0033890*, *HD.06G0019520*, *HD.12G0014330)*. One *MYB (HD.03G0018510)* and one *WRKY (HD.03G0017510)* TFs were identified in the pink module. Therefore, the expression of *MYB 44 (HD.06G0033760)* and *WRKY 17 (HD.12G0001660)* in L4 treatment negatively regulated the content of EGCG.

### 2.11. Verification of the Expression Pattern of DEGs

**Figure 11:**
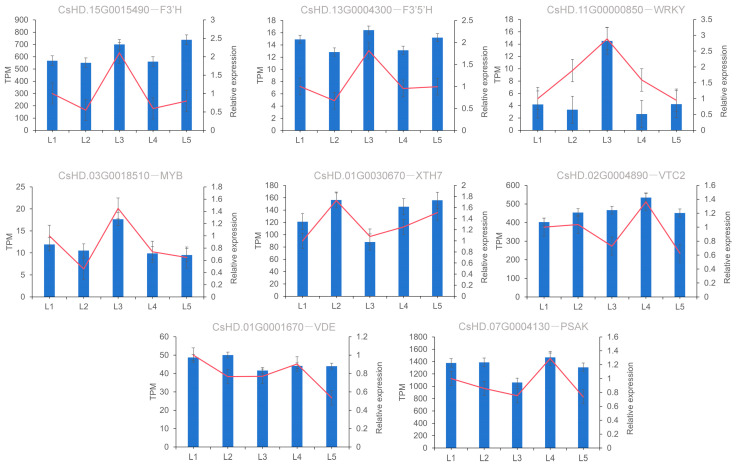
Expression level verification of eight DEGs. Notes: The x-axis represents different treatments (L1–L5: 0, 0.15, 0.45, 0.6, and 0.9 mmol/L mg^2+^, respectively) and the y-axis represents relative expression. The data represent the means from three replicates with three biological repeats. Error bars indicate SE. Red line represents the amount of gene expression in the transcriptomic.

In order to verify the reliability of RNA-seq data, the expression levels of eight DEGs were detected by qRT-PCR, including two DEGs related to EGCG biosynthesis, two TFs involved in the synthesis pathway, and four randomly selected DEGs. As shown in Figure 11, the gene expression trends of qRT-PCR and RNA-seq were basically the same, indicating that the DEGs obtained based on transcriptome data in this study were credible.

## 3. Discussion

EGCG is a key component of catechins with physiological activity and wide-ranging applications. It is one of the crucial factors influencing the quality of tea. Notably, EGCG cannot be synthesized artificially. In this study, different concentrations of magnesium nutrients were used to process tea tree sprouts, and a combination of high-throughput and high-sensitivity metabolomics and transcriptomics methods were used to understand the role of magnesium nutrients in regulating EGCG biosynthesis. Magnesium deficiency is a prevalent issue in tea gardens, particularly in areas where chemical fertilizers are excessively or inadequately used [17]. Previous studies have demonstrated that the accumulation of catechins is generally influenced by factors such as temperature, light, soil water content, moisture, endogenous hormones, and nutrition [18,19,20]. The biosynthesis of EGCG and its regulatory mechanisms have been extensively documented [21]. However, the way in which magnesium nutrition regulates the biosynthesis of EGCG has not yet been reported.

### 3.1. Magnesium Treatment Affected the Characteristics, Metabolic Components, and Quality of Tea Plants

Tea plants are rich in characteristic secondary metabolites, including polyphenols, purine alkaloids, amino acids, and aromatic compounds. Among them, catechins, theanine, caffeine, and other substances can make tea have a strong and wide range of healthcare functions in addition to a charming aroma and pleasant taste. Catechins are representative secondary metabolites of tea plants, which determine the unique flavor and health benefits of different teas [21]. In recent years, relevant studies have also revealed that magnesium nutrition has an important impact on the caffeine, polyphenols, theanine, etc., in tea. In this study, the quantitative analysis of catechins, free amino acids, and tea polyphenols in the shoots of tea plants at five different Mg^2+^ concentrations showed that the EGCG content of tea plants increased significantly when the tea plants were watered with 0.15 and 0.45 mmol/L treatments, but decreased in 0.6 and 0.9 mmol/L treatments, which may be due to the fact that the application of the Mg^2+^ fertilizer helped increase the tea yield and reduce the ratio of total polyphenols to amino acids in tea, but under the regulation of glutamine synthetase 1.1 (*CsGS1.1*), nitrogen assimilation was enhanced, so the EGCG content was decreased under the regulation of Mg^2+^ 0.6 mmol/L [22,23,24]. After the experimental treatment, the contents of the six identified catechins showed a gradual decreasing trend. With the increase in magnesium concentration, the content of tea polyphenols fluctuated, and the content of free amino acids was higher than that of the control (L1: 0 mmol/L). It can be seen that an appropriate increase in magnesium nutrition is conducive to the increase in catechins, caffeine, tea polyphenols, and free amino acids. With the increase in magnesium concentration, the chlorophyll content of tea tree increased first and then decreased. An appropriate magnesium concentration (0.15 mmol/L) could increase the chlorophyll content of tea plants; magnesium deficiency and excessive magnesium content reduced the chlorophyll content of tea plants, which is consistent with the results of previous studies [25]. Thus, it was speculated that magnesium affected the chlorophyll content of tea plants, and then affected the growth of tea plants.

### 3.2. Magnesium Treatment Regulates Metabolites in EGCG Biosynthesis

Changes in magnesium concentration affect the content of flavonoids and phenolic acids, and play an important role in the biosynthesis of EGCG. In this study, KEGG enrichment analysis was performed on the SCMs of the two comparison groups (L2 vs. L1, L3 vs. L1, L4 vs. L1, and L5 vs. L1), and it was found that the SCMs in each comparison group were mainly enriched in flavonoids and phenylpropanoid biosynthesis pathways. Under the conditions of low magnesium, the activity of the flavonoid synthesis pathway was enhanced, which may be because the low-magnesium environment reduces competition with the substrate of the phenylalanine and flavonoid pathways and thus promotes the accumulation of flavonoid substances [26,27]. However, higher concentrations of magnesium restricted the shikimic acid and phenylalanine pathways, thus retaining more substrates [28]. The highest proportion of organic acids in L2 treatment may be due to the accumulation of gallic acid as a precursor of catechins and methyl gallate, which affects the biosynthesis of EGCG [29,30]. Therefore, it is speculated that the metabolic flow of flavonoid pathways is preferentially assigned under low-magnesium conditions, which promotes the accumulation of EGCG.

### 3.3. Magnesium Treatment Regulates the Expression of Key Genes in EGCG Biosynthesis

Magnesium nutrition not only affects the content of metabolites, but also affects the synthesis of EGCG by regulating the expression of key genes. In this study, a total of 43 key genes involved in EGCG biosynthesis were annotated. Among them, 19 structural genes are involved in the phenylpropanoid pathway, 20 structural genes are involved in the flavonoid pathway, and 4 structural genes are involved in the shikimic acid pathway. In the phenylalanine pathway, the expression of the *CsPAL* gene is up-regulated under L2 and L3 treatments, which is the same trend as EGCG, and may be a key gene involved in the regulation of EGCG biosynthesis. The expression of the CsC4H gene was up-regulated under L3 treatment, which may regulate the accumulation of p-coumaric acid expression and promote EGCG biosynthesis [31]. In the flavonoid pathway, the CsF3′H and CsDFR genes are up-regulated at L3, while leucocyanidin, eriodictyol, and dihydromyricetin contents are decreased, suggesting that the up-regulated expression of these genes may not directly promote the synthesis of EGCG, which may be related to the enhancement in other secondary metabolic branches [27]. Therefore, it is hypothesized that different concentrations of magnesium treatment significantly affect the expression of genes involved in EGCG biosynthesis.

### 3.4. Transcription Factors Play an Important Role in EGCG Biosynthesis

Transcription factors are regulated by binding to specific cis-elements in the promoter region, and the expression of target genes plays an important regulatory role in plant growth, metabolism, and stress responses. In this study, four modules were screened based on the WGCNA method. The key transcription factors in the four modules were functionally annotated, and it was found that they were mainly involved in the regulation of plant secondary metabolism and the regulation of the plant growth process. A study of genetic changes in tea plants under nitrogen, phosphorus, and potassium deficiency conditions suggests that *WRKY*, *MYB*, and members of the bHLH family may regulate the biosynthesis of the catechins, theanine, and caffeine [32], *MYB*, *bHLH*, *WRKY*, *NAC* are the main families involved in EGCG biosynthesis [31]; *WRKY*, *C2H2*, *C3H*, *NAC*, and *ERF* transcription factors are associated with biological and abiotic resistance genes, and are thought to be highly correlated with catechin pathway genes [33]; and the *MYB-bHLH-WD40* complex (MBW complex) is involved in the transcriptional regulation of downstream structural genes of the flavonoid biosynthesis pathway [34]. Among the annotated transcription factors, four *MYB* transcription factors *(HD.04G0021130*, *HD.06G0008880*, *HD.03G0018510*, *HD.06G0033760)* regulated catechin metabolism in tea tree after magnesium treatment, which is consistent with the results of previous studies, suggesting that *CsMYB* may be a key gene regulating EGCG biosynthesis in tea plants. The expression levels of three *WRKY* transcription factors *(HD.03G0017510*, *HD.01G0021500*, *HD.11G0000850)* were significantly up-regulated under L3 (0.45 mmol/L) treatment, suggesting that these four transcription factors play a regulatory role in EGCG biosynthesis. Therefore, combined with the results of this study, it is speculated that magnesium may enhance the activity of the flavonoid pathway and promote EGCG synthesis in a low-magnesium environment by regulating the relationship between the flavonoid pathway and metabolic flux through transcription factors.

## 4. Materials and Methods

### 4.1. Tea Plant Materials

The experiment was completed in March 2023 at Anxi County, Fujian Province (25°26′ N, 118°17′ E), Fujian Agriculture and Forestry University. One-year-old ‘Huangdan’ cutting tea seedlings with healthy, disease-free, and consistent growth were selected as test materials and planted in pots filled with river sand (48 × 27 × 15.5 cm). Each treatment was set up with 15 pots, totaling 75 pots, with each pot containing 10 tea seedlings. These seedlings were treated with nutrient solution containing different concentrations of Mg^2+^ (L1–L5: 0, 0.15, 0.45, 0.6, 0.9 mmol/L, respectively), and the L1 treatment was used as the control group. The nutrient solution was improved according to the formula of Xiao Xi Maoyi: 1 mmol/L of (NH_4_)_2_SO_4_, 0.5 mmol/L of Ca(NO_3_)_2_; 1.0 mmol/L of K_2_SO_4_, 0.6 mmol/L of MgSO_4_, 1.0 mmol/L of NH_4_H_2_PO_4_; 0.4 mmol/L of Al_2_(SO_4_)_3_, 46 μmol/L of H_3_BO_3_, 9 μmol/L of MnSO_4_; 2 μmol/L of CuSO_4_, 9 μmol/L of ZnSO_4_, 2.6 μmol/L of Na_2_MoO_4_; and 30 μmol/L of Fe-EDTA (C_10_H_12_FeN_2_NaO_8_). Solutions of 0.01 mol/L of HCL and 0.01 mol/L of NaOH were used to adjust the pH of the nutrient solution. The nutrient solution and water were distributed in proportion, and each pot was watered 500 mL each time and three times a week. In May 2023, one bud and two leaves were collected as materials in the five treatments for experimentation: a portion of the sample was dried and stored at −20 °C in the refrigerator (drying at 120 °C for 60 min) for subsequent physiological and biochemical component testing. The other part was immediately frozen with liquid nitrogen and stored in a −80 °C freezer for transcriptomics and metabolomics testing.

### 4.2. Determination of the Main Biochemical Components

The catechins, caffeine, and GA fractions were determined, and the test reagents and instruments were determined according to the ultra-performance liquid chromatography–quadrupole time-of-flight/mass spectrometer (UPLC-Q-TOF/MS) method by Lin et al. [35]. The determination of tea polyphenols refers to GB/T 8313-2018 [36] (forinphenol colorimetric method), and the determination of free amino acids refers to GB/T 8314-2013 [37]. The chlorophyll content was extracted by ground alcohol. The magnesium content in tea leaves was determined with reference to GB 5009.241-2017 [38] (atomic absorption spectrophotometry). Three replicates were performed for each treatment after sampling.

### 4.3. Analysis of Non-Volatile Metabolites by Widely Targeted Metabolomics

Specific analyses of broad-spectrum metabolites were performed according to previously published methods [39], and they were dried in a MetWare freeze-drying chamber (Wuhan, China). Ultra-performance liquid chromatography–dual mass spectrometry (UPLC-MS/MS) analysis (Applied Biosystems 4500 QTRAP, tandem mass spectrometry from Thermo Fisher Scientific, Waltham, MA, USA) was used and performed according to the method of the previous study: an Agilent SB-C18 column (Agilent Corporation, Santa Clara, CA, USA) was used with mobile phase A including 0.1% formic acid in ultrapure water, and mobile phase B with acetonitrile. The elution gradient was set as follows: initially, phase B was proportionally set to 5%, and then linearly to 95% over 9 min, and then at 95%. This was held for 1 min, and then phase B fell to 5% within 10~14 min and kept balanced. The flow rate was 0.35 mL·min^−1^, the column temperature was 40 °C, and the injection volume was 2 μL. Mass spectrometry was performed using ESI, ion source gas I (GSI), and ion source gas II (GSII) at 50 psi, 60 psi, and with the MRM mode, respectively, by scanning and optimizing the declustering voltage (DP) and collision energy (CE). The optimal DP and CE values were determined for each MRM transition [40].

The data obtained using the UPLC-MS/MS method described above were processed using Analyst 1.6.3 software. Principal component analysis (PCA), hierarchical cluster analysis (HCA), and discriminant analysis with orthogonal latent structure projection (OPLS-DA) were used to compare and analyze different metabolites. In this study, the screening criteria for significantly changed metabolites (SCMs) were VIP ≥ 1.0, fold change ≥ 1.2 or fold change ≤ 0.83.

### 4.4. Transcriptomics Analysis

Using the Shanghai Major Bio-pharm Technology Co., Ltd. sequencing platform, all mRNAs transcribed in specific tissues or cells of eukaryotic organisms over a specified period were sequenced. Sequencing experiments were performed using Illumina’s Truseq^TM^ RNA sample prep (CA, USA) kit for library construction. Total RNA was isolated from tissue samples, and the concentration and purity of isolated RNA were determined using Nanodrop2000. RNA integrity was confirmed by agarose gel electrophoresis. Transcriptome data were analyzed as per the previous methods [41], in which the transcriptome library was compared to the filtered sequences to the ‘HuangDan’ tea tree genome (*HD*) using Illumina’s TruSeqTM RNA sample preparation kit using TopHat 2 (a popular spliced aligner for RNA-sequence (RNA-seq) experiments) software [42]. RSEM 1.3.3 software (RNA-seq by expectation–maximization: https://deweylab.biostat.wisc.edu/rsem/, access on 28 October 2023) was used to analyze the expression levels of genes in the samples, which were calculated using the TPM (transcripts per million) method. Gene differential expression analysis was performed between the two groups using DESeq2 v 1.22.1 software, and the *p* value was corrected using the Benjamini–Hochberg method [43]. DEGs were defined as |log2 foldchange| ≥ 1 and *p* < 0.05.

### 4.5. Redundancy Analysis

Redundancy analyses (RDAs) were performed to explore the potential correlation between metabolite levels and synthetic genes in the EGCG biosynthesis pathway through metabolomics and transcriptomics data. Specifically, 20 flavonoid content levels and the expression levels of 43 hub genes within the EGCG synthesis pathway after treatment with magnesium were imported into Canoco 5.0 for RDA, utilizing the default parameters.

### 4.6. Co-Expression Analysis

Based on the genome of ‘Huangdan’ [42], TFs were annotated and weighted gene co-expression network analysis (WGCNA) was performed. The co-expression network was constructed to identify key modules based on the topological overlap matrix (TOM) with the soft threshold power set to 5, the minimum module size set to 30, and the merging threshold for similar modules set to 0.5. Gene modules highly correlated with metabolites (TPM > 1, cv > 0.1) were identified from the screening data. Co-expression networks were constructed by calculating Pearson correlations using the abundance of 46,053 genes and 20 metabolites from the screening data. Central co-expression modules were visualized using Cytoscape 3.9.0 software.

### 4.7. Validation of Quantitative Real-Time PCR

In order to verify the accuracy of the transcriptome, 8 DEGs were selected for expression level verification. Using Primer 3 plus (HTTPS: https://www.bioinformatics.nl/cgi-bin/primer3plus/primer3plus.cgi, accessed on 8 January 2024) design primers from their website, the primer sequences are shown in Table 2. The *CsGAPDH (GE651107)* gene was used as the internal reference gene, and the 2^−ΔΔCt^ method was used to quantify the expression of related genes. cDNA synthesis and q-PCR detection were performed according to previous methods [44]. All samples were analyzed in three biological replicates.

### 4.8. Statistical Analysis

The tests were repeated in triplicate, and the result of each test is expressed as the average of three replicates. SPSS 26.0 software was used for statistical analysis of the data (*p* < 0.05), and GraphPad Prism 8.0 and TBtools-II were used to generate path maps, heat maps, and network maps.

## 5. Conclusions

In this study, we discussed the effects of different treatments on EGCG synthesis. Quantitative analysis showed that treatment with an appropriate magnesium concentration (0.15 mmol/L in this test) could promote the biosynthesis of EGCG in tea plants, with an increase in magnesium concentration inhibiting the biosynthesis of EGCG in tea plants. Metabolomics clearly showed the accumulation of flavonoids and phenolic acids; the accumulation of metabolites in the EGCG synthesis pathway was especially notable. Among them, the structural genes *CsPAL (HD.01G0005520*, *HD.02G0024350)*, *Cs4CL (HD.15G0008250*, *HD.13G0010220)*, *CsDFR (HD.04G0026220)*, *CsANS(HD.12G0016700)*, and *CsaroDE (HD.03G0002480)* had positive regulatory effects on the biosynthesis of EGCG, while *CsPAL (HD.13G0009900*, *HD.06G0008610)*, *CsC4H (HD.06G0017130)*, *Cs4CL (HD.02G0027390*, *HD.04G0003270)*, *CsCHS (HD.10G0022640)*, *CsCHI (HD.01G0011100)*, *CsF3′H (HD.15G0015490)*, *CsF3′5′H (HD.13G0004300)*, *CsANS (HD.07G0023630)*, *Csaro B (HD.01G0028400)*, *CsSCPL (HD.01G0041070)*, TFs *MYB 44 (HD.06G0033760)*, and *WRKY 17 (HD.12G0001660)* had negative regulatory effects on the biosynthesis of EGCG. Further research should focus on the following two areas: (1) What is the effect of the interaction of magnesium with other nutrients on EGCG biosynthesis? (2) What is the mechanism of EGCG biosynthesis under the influence of magnesium, which can be uncovered using translational and proteomic techniques?

## Figures and Tables

**Table 1:** Contents of caffeine, gallic acid, catechin fractions, tea polyphenols, and free amino acids in tea shoots treated by magnesium (mg/g).

Compound	Treated Magnesium Concentration (mmol/L)				
	0	0.15	0.45	0.6	0.9
Gallic acid (GA)	1.69 ± 0.03 ab	1.78 ± 0.12 a	1.71 ± 0.02 ab	1.66 ± 0.02 b	1.67 ± 0.05 b
Caffeine (CA)	5.18 ± 0.20 a	4.29 ± 1.85 a	4.99 ± 0.48 a	4.92 ± 0.14 a	4.71 ± 0.20 a
Catechin (C)	1.55 ± 0.03 a	1.45 ± 0.05 b	1.47 ± 0.09 b	1.42 ± 0.19 b	1.34 ± 0.02 c
Epicatechin (EC)	1.59 ± 0.38 a	1.59 ± 0.12 a	1.52 ± 0.39 a	1.50 ± 0.27 a	1.49 ± 0.08 a
Epigallocatechin gallate (EGCG)	60.84 ± 2.72 ab	63.82 ± 2.19 a	63.56 ± 1.33 ab	56.87 ± 4.67 ab	59.12 ± 3.36 b
Epigallocatechin (EGC)	48.80 ± 4.17 ab	52.74 ± 2.43 ab	54.68 ± 1.69 a	45.76 ± 2.47 b	47.34 ± 1.88 ab
Gallocatechin (GC)	23.31 ± 0.7 ab	14.68 ± 0.2 a	26.91 ± 1.22 a	19.95 ± 3.38 bc	23.91 ± 1.29 ab
Epicatechin gallate (ECG)	14.14 ± 0.48 b	27.01 ± 0.89 a	14.39 ± 0.29 b	13.07 ± 0.47 b	13.85 ± 0.55 b
Total catechins	151.96 ± 6.47 ab	163.11 ± 5.61 a	164.27 ± 4.48 a	140.29 ± 3.93 b	153.767 ± 3.12 ab
Tea polyphenols	12.12 ± 0.62 c	12.73 ± 0.33 bc	12.30 ± 0.29 bc	13.82 ± 0.62 a	13.78 ± 0.14 a
Free amino acids	0.63 ± 0.022 ab	0.69 ± 0.099 a	0.62 ± 0.037 ab	0.63 ± 0.015 ab	0.60 ± 0.049 ab
Phenolammonia ratio	19.28 ± 1.54 bc	18.77 ± 3.08 bc	20.63 ± 0.85 b	22.12 ± 0.98 ab	23.14 ± 2.11 ab

Notes: Data are expressed as “mean ± standard deviation”; different lowercase letters indicate significant differences between treatments (*p* < 0.05).

**Table 2:** Primer sequences.

Gene Name	Upstream Primer (5′-3′)	Downstream Primers (5′-3′)
*CsHD.15G0015490-F3′H*	GCCCAATGCTGATGTTAGGG	ATGGACCAAGGTCGCAGTTA
*CsHD.13G0004300-F3′5′H*	TTTCTCGACGTTGTGATGGC	CCCATTCAACTGTGCTTGCT
*CsHD.11G0000850-WRKY*	GAGCCGAGATTTGCGTTCAT	TTTGGCCGTACTTCCTCCAT
*CsHD.03G0018510-MYB*	AGGCTCATGGAGAAGGCAAT	TCATCCACCTTAGCCTGCAA
*CsHD.02G0004890-XTH7*	ACGAAGCAAGAGGAATCCCA	CAGTCATCGGCTTCCCAAAG
*CsHD.02G0004890-VTC*	AGAGGCCGACTGAGTTTCAA	GCATTCCGGAAGAACTGGAC
*CsHD.01G0001670-VDE*	ACGCATGGGATGGATATGGT	CTCCAGTCTCTCCACAAGGG
*CsHD.07G0004130-PSAK*	AGCAAACAGGAAAGCCACAG	AGAGCCACAAGCCAAGGTAT

## Data Availability

Data will be made available on request.

## Supplementary Materials

The following supporting information can be downloaded at: https://www.mdpi.com/article/10.3390/plants14050684/s1. Table S1: Major differential in common metabolites between L2 and L1 treatments under Mg regulation. Table S2: Major differential in common metabolites between L3 and L1 treatments under Mg regulation. Table S3: Major differential in common metabolites between L4 and L1 treatments under Mg regulation. Table S4: Major differential in common metabolites between L5 and L1 treatments under Mg regulation. Table S5: Expression of genes related to EGCG biosynthesis metabolism in tea tree after treatment with different concentrations of magnesium.
