# Supplementary material for: Metabolome and Transcriptome Analysis Reveals the Regulatory Effect of Magnesium Treatment on EGCG Biosynthesis in Tea Shoots (Camellia sinensis)
Source: Plants (Basel). 2025 Feb 23;14(5):684. doi: 10.3390/plants14050684 (PMC11901942; doi:10.3390/plants14050684)
Supplement: Supplementary file 1 [file plants-14-00684-s001.zip › plants-3423875-supplementary.pdf]

Supplementary Materials

Table S1: Major differential in common metabolites between L2 and L1 treatments under Mg regulation

Rank	Compounds	Formula	CAS	L2 ()	L1
1	Punicic acid (9Z,11E,13Z-octadecatrienoic acid)	C18H30O2	544-72-9	2681380.811	618432.2134
2	9S-Hydroperoxy-10E,12Z-octadecadienoic acid	C18H32O4	5502-91-0	145911.9334	53955.81707
3	1-(2,3-dihydroxypropoxy)-3-(((2-(dimethylamino)ethoxy)(hydroxy) phosphoryl)oxy)propan-2-yl (E)-hexadec-9-enoate	C26H52NO9P	-	13408.91398	6225.849617
4	2-Aminohexadecane-1,16,16-triol	C16H35NO3	-	914471.8688	364588.3942
5	LysoPC 20:0	C28H58NO7P	108341-80-6	90030.71713	26323.29446
6	2-Aminohexadecane-1,5,6-triol	C16H35NO3	-	127795.4428	52298.23104
7	Glucosyl 2,3-Dihydroxy-2-Methylbutanoic Acid	C11H20O9	-	2295489.234	943352.6649
8	3-Ureidopropionic Acid	C4H8N2O3	462-88-4	79269.27786	38490.32786
9	8(26),14(27)-onoceradiene-3β,21α-diol	C31H52O2	-	98274.37383	47757.9752
10	6-Hydroxy-lupane-20(29)-en-3-one-28-oic acid	C30H46O4	-	131706.3889	55353.09977
11	3-Hydroxyolean-12-ene-27,28-dioic acid (Cincholic acid)	C30H46O5	5948-32-3	50551.96181	20782.64037
12	2,3,19-Trihydroxy-24-oxo-olean-12-en-28-oic acid	C30H46O6	-	57314.21636	25869.11868
13	Vanillylamine	C8H11NO2	1196-92-5	503825.4812	220375.737
14	3,4-Dimethylellagic acid 4'-sulfate	C16H10O11S	-	135687.599	39460.26075
15	Gallic acid-1-O-xyloside	C12H14O9	-	1159101.18	35129.40523
16	2,5,8-trimethyldeca-2,4,7-triene-1,10-diol	C13H22O2	-	1743604.467	653802.6678

Continued table S1: Major differential in common metabolites between L2 and L1 treatments under Mg regulation

Rank	Compounds	Formula	CAS	L2	L1
17	Manninotriose	C18H32O16	13382-86-0	32513.26068	13783.77133
18	Secoisolariciresinol 4-O-glucoside	C26H36O11	-	1416347.926	634852.423
19	Isolariciresinol-9'-O-rhamnoside (Aviculin)	C26H34O10	156765-33-2	16821.89242	7243.009889
20	1-Hydroxypinoresinol-4'-O-Glucoside	C26H32O12	-	72557.17848	30239.256
21	Quercetin-3-O-glucuronide	C21H18O13	22688-79-5	571344.1604	251998.3017
22	Kaempferol-3-O-(2'''-p-Coumaroyl)sophoroside-7-O-Glucoside	C42H46O23	-	49875.203	16788.96033
23	Chrysoeriol-7-O-rutinoside-5-O-glucoside	C34H42O20	-	46479.8725	20461.37967
24	Guanine	C5H5N5O	73-40-5	57153649.8	27960944.63
25	Elaidolinolenic acid	C18H30O2	28290-79-1	4917232.232	1026348.041
26	2-Hydroxyphenol-1-O-glucosyl(6→1)rhamnoside	C18H26O11	-	21041.80644	9927.131333
27	Dihydroxybenzoyl xyloside	C12H14O8	-	339146.8987	26263.07633
28	Isomartynoside	C31H40O15	94410-22-7	13410563.34	4748238.297
29	Salicin 6'-Sulfate	C13H18O10S	-	4579015.188	141299.7203
30	4-Hydroxyphenyl 6-O-(2-Methyl-3-Hydroxypropionyl)-Beta-D-Galactopyranoside	C16H22O9	-	405043.6403	155014.3454
31	Methyl 3-(3-hydroxy-4-methoxyphenyl)propanoate	C11H14O4	129150-61-4	45742.87717	22405.104
32	Tranexamic Acid	C8H15NO2	1197-18-8	2358460.908	5048633.069
33	Jasmonic acid	C12H18O3	77026-92-7	259307.2651	591098.167
34	Cis-Aconitic acid	C6H6O6	585-84-2	3120738.401	6365309.111
35	Lupa-1,20(29)-dien-3-one (Glochidone)	C30H46O	-	409893.7026	903093.9687
36	Pipecolic acid	C6H11NO2	535-75-1	10107516.35	21955798.82
37	Castanoside B[Kaempferol-3-O-(4'',6''-di-p-coumaroyl)mannoside]	C39H32O15	-	728564.6422	1539942.058

Continued table S1: Major differential in common metabolites between L2 and L1 treatments under Mg regulation

Rank	Compounds	Formula	CAS	L2	L1
38	3'-O-Methyl-6-hydroxygallocatechin 3-O-(N-Ethylglutamine ester) 3'-Gallate	C30H32N2O14	-	964231.603	2407557.842
39	3'-O-Methylgallocatechin 3-O-(N-Ethylglutamine ester) 4'-Gallate	C30H32N2O13	-	434290.5887	1140569.119
40	5,6,3',4'-Tetrahydroxy-3,7-dimethoxyflavone-6-O-glucoside	C23H24O13	-	1597754.212	3258205.42
41	Limocitrin 3-Glucoside	C23H24O13	-	1597754.212	3258205.42
42	4'-O-Methylgallocatechin 3-O-(N-Ethylglutamine ester) 3'-Gallate	C30H32N2O13	-	47914.55511	111976.0868
43	3-Dehydroshikimic Acid 5-(3-Methylsalicyloyl)Gallate	C22H18O11	-	3831567.479	9449670.668
44	3-Dehydroshikimic Acid 5-(3-Vanilloyl)Gallate	C22H18O12	-	6231757.909	14925542.59
45	1-O-Galloyl-6-O-p-Coumaroyl-β-D-glucose	C22H22O12	-	80439.56433	180374.8123
46	L-Theanine	C7H14N2O3	3081-61-6	1158562.971	2504500.828
47	L-Gamma-Glutamyl-L-Theanine	C12H21N3O6	-	637674.162	1351751.376

Table S2: Major differential in common metabolites between L3 and L1 treatments under Mg regulation

Rank	Compounds	Formula	CAS	L3	L1
1	Punicic acid (9Z,11E,13Z-octadecatrienoic acid)	C18H30O2	544-72-9	3295378.031	618432.2134
2	9S-Hydroperoxy-10E,12Z-octadecadienoic acid	C18H32O4	5502-91-0	263172.7082	53955.81707
3	2-Aminooctadecane-1,16,18,18-tetraol	C18H39NO4	-	185268.0548	70224.37546
4	LysoPC 20:0	C28H58NO7P	108341-80-6	69976.07777	26323.29446

Continued table S2: Major differential in common metabolites between L3 and L1 treatments under Mg regulation

Rank	Compounds	Formula	CAS	L3	L1
5	Glycerol 9(E),11(Z),13(E)-octadecatrienoyl ester	$C_{21}H_{36}O_4$	-	730211.8079	1823370.478
6	Monolinolenin*	$C_{21}H_{36}O_4$	75685-85-7	725115.9592	1784700.806
7	2-α-Linolenoyl-glycerol*	$C_{21}H_{36}O_4$	55268-58-1	725527.9822	1791423.768
8	13(s)-hydroperoxy-(9z,11e,15z)-octadecatrienoic acid	$C_{18}H_{30}O_4$	67597-26-6	2812957.406	5767964.271
9	1-α-Linolenoyl-glycerol*	$C_{21}H_{36}O_4$	-	784148.379	2206562.042
10	1-Monolinolenoyl-Rac-Glycerol	$C_{21}H_{36}O_4$	18465-99-1	871657.0084	2024479.675
11	Piperonylic acid	$C_8H_6O_4$	94-53-1	994157.5602	459816.2956
12	Glucosyl 2,3-Dihydroxy-2-Methylbutanoic Acid	$C_{11}H_{20}O_9$	-	2444333.434	943352.6649
13	Jasmonic acid	$C_{12}H_{18}O_3$	77026-92-7	239810.2632	591098.167
14	Methyl abieta	$C_{21}H_{32}O_2$	-	467781.7327	130802.808
15	8(26),14(27)-onoceradiene-3β,21α-diol	$C_{31}H_{52}O_2$	-	211375.34	47757.9752
16	2,3,19,23-Tetrahydroxyurs-12-en-28-oic acid	$C_{30}H_{48}O_6$	-	1889763.934	865463.3234
17	celastolide	$C_{30}H_{46}O_5$	-	10488361.84	3879348.701
18	orthosphenic acid	$C_{30}H_{48}O_5$	86632-20-4	1773595.932	841075.1489
19	2,19-Dihydroxy-3-oxours-12-en-28-oic acid	$C_{30}H_{46}O_5$	176983-21-4	837517.3434	302795.4592
20	Kadcoccinic acid G	$C_{30}H_{46}O_5$	1802768-42-8	60005.81052	21081.53642
21	Cannabifolin C	$C_{30}H_{48}O_5$	-	441570.3763	194018.7189
22	Clerodendrin I	$C_{33}H_{44}O_{14}$	-	538378.9706	167409.5647
23	6-Hydroxy-lupane-20(29)-en-3-one-28-oic acid	$C_{30}H_{46}O_4$	-	179600.5624	55353.09977
24	3,19,23-Trihydroxyurs-12-en-28-oic acid (Rutundic acid)	$C_{30}H_{48}O_5$	20137-37-5	41369.1861	19588.95864
25	Blumenol C	$C_{13}H_{22}O_2$	-	2874365.622	1415445.854

Continued table S2: Major differential in common metabolites between L3 and L1 treatments under Mg regulation

Rank	Compounds	Formula	CAS	L3	L1
26	11,12-epoxy-13-hydroxy-3-Oxooleanane-28-oic acid gamma-lactone (Liquidambaric Lactone)	C30H44O4	185051-75-6	493929.3294	200511.5929
27	2,3,19,23,24-Pentahydroxyolean-12-en-28-oic acid	C30H48O7	-	48573.91608	8468.77145
28	1'S,4'S-4'-Dihydroabscisic acid-4'-O-β-malonylglucopyranoside	C24H34O12	-	177318.4611	67543.017
29	Blumenol C glucoside	C19H32O7	135820-80-3	928861.4852	211665.3785
30	Cordianol B	C30H48O5	-	443817.637	216899.7019
31	Javanicolide C	C26H36O11	-	1804419.275	808941.8657
32	dihydromachaeric acid lactone	C30H48O3	-	749382.3465	319214.0931
33	3S-3-hydroxy-β-damascon	C13H20O2	-	373455.7001	166566.8857
34	2,3-Dihydroxyoleana-11,13(18)-dien-28-oic acid (Camaldulenic acid)	C30H46O4	71850-15-2	400223.7888	180852.4727
35	Lup-12-en-15α,19β-diol-3,11-dioxo-28-oic acid	C30H44O6	-	51392.69377	22619.20867
36	3-Hydroxy-7,8-dehydro-ionol-9-O-β-D-glucopyranoside	C19H32O7	-	489224.0989	243727.0967
37	3,4-Open loop-lupine-4(23),20(29)-diene-24-hydroxy-3-carboxylic acid	C30H48O3	-	2808460.733	1097433.164
38	3,4-Cycloopen-cycloaltin-4(29),25-diene-24(S)-hydroxy-3-carboxylic acid	C30H48O3	-	2644755.947	994531.7145
39	2,3,19-Trihydroxy-24-oxo-olean-12-en-28-oic acid	C30H46O6	-	79365.17189	25869.11868
40	Genipin	C11H14O5	6902-77-8	386852.6453	126212.2242
41	Dehydroxyampelopsisionoside	C19H32O7	-	463000.4859	201367.2342
42	2,3,6-Trihydroxyurs-12-en-28-oic acid (Madasiatic acid)	C30H48O5	26532-66-1	435968.8571	199683.0612
43	Lupa-1,20(29)-dien-3-one (Glochidone)	C30H46O	-	433376.6541	903093.9687
44	14-hydroxy-14-(hydroxymethyl)-5,9-dimethyltetracyclo [11.2.1.01,10.04,9]hexadecane-5-carboxylic acid	C20H32O4	-	275757.9765	625476.2701

Continued table S2: Major differential in common metabolites between L3 and L1 treatments under Mg regulation

45	3-pyridine-methanol-O-β-D-glucopyranosyl	C12H17NO6	-	8835907.257	4269354.601
46	Tetraphyllin A	C12H17NO6	-	8182783.254	3931975.705
47	N-Feruloylputrescine N-glucoside	C20H30N2O8	-	842258.857	261224.4198
48	Indole-5-carboxylic acid*	C9H7NO2	1670-81-1	20356.07842	9970.60975
49	Pipecolic acid	C6H11NO2	535-75-1	6412856.895	21955798.82
50	2-Phenylethylamine	C8H11N	156-28-5	433067.5582	1033486.615
51	3,4-Dimethylellagic acid 4'-sulfate	C16H10O11S	-	323573.9957	39460.26075
52	Gallic acid-1-O-xyloside	C12H14O9	-	2805161.26	35129.40523
53	3-Ethyl-7-hydroxyphthalide	C10H10O3	-	1827921.648	723072.713
54	Roseoside	C19H30O8	54835-70-0	360690.637	152509.0787
55	2,5,8-trimethyldeca-2,4,7-triene-1,10-diol	C13H22O2	-	2121308.838	653802.6678
56	Syringaldehyde; 4-Hydroxy-3,5-Dimethoxybenzaldehyde	C9H10O4	134-96-3	388709.5682	167427.3233
57	[(2R,3R,4S,5S)-4-hydroxy-5-(hydroxymethyl)-2-[[(2R,3S,4S,5R,6S)-3,4,5-trihydroxy-6-[2-[(1R)-4-methylcyclohex-3-en-1-yl]propan-2-yloxy]oxan-2-yl]methoxy]oxolan-3-yl]3,4,5-trihydroxybenzoate	C28H40O14	-	112285.808	37180.40239
58	Alangionoside L	C19H32O7	-	489224.0989	243727.0967
59	Manninotriose	C18H32O16	13382-86-0	36571.99373	13783.77133
60	Moniliferanone D	C22H32O4	-	6272963.958	13649356.47
61	Squamocin K	C35H62O6	161169-70-6	34614.20596	145173.3464
62	Clovanmagnolol	C33H42O3	-	98081500.8	45958309.96
63	Methoxy-erythro-Guaiacyl glycerol β-threo-syringyl glycerol ether	C22H30O11	-	408156.5972	177899.9962
64	Secoisolariciresinol 4-O-glucoside	C26H36O11	-	1431401.919	634852.423

Continued table S2: Major differential in common metabolites between L3 and L1 treatments under Mg regulation

Rank	Compounds	Formula	CAS	L3	L1
65	Isolariciresinol-9'-O-rhamnoside (Aviculin)	C26H34O10	156765-33-2	16357.02333	7243.009889
66	6-((4-(3-hydroxy-2-(4-(3-hydroxypropyl)-2-methylphenoxy)propoxy)-2-methoxyphenoxy)methyl)tetrahydro-2H-pyran-2,3,4,5-tetraol	C26H36O11	-	1538447.542	713634.1545
67	1-Hydroxypinoresinol-4'-O-Glucoside	C26H32O12	-	66243.34696	30239.256
68	Matairesinol-4'-O-glucoside (Matairesinoside)	C26H32O11	23202-85-9	1455511.791	399987.5247
69	Isolariciresinol	C20H24O6	548-29-8	180185.157	80759.692
70	Medioresinol	C21H24O7	40957-99-1	9500.832639	25230.97944
71	Dehydrodiconiferyl alcohol	C20H22O6	4263-87-0	303070.3651	622513.0667
72	4,8-Dihydroxy Naphthol-1-O-glucoside	C16H18O8	-	3078449.809	1494705.117
73	Phloretin-2'-O-glucoside (Phlorizin)	C21H24O10	60-81-1	3552391.7	1563930.415
74	Phloretin-4'-O-glucoside (Trilobatin)	C21H24O10	4192-90-9	4159501.872	1424808.792
75	Dihydrocharcone-4'-O-glucoside	C21H24O10	-	4051225.683	1415653.995
76	8,11-dimethoxy-2h-[1,3]dioxolo[4,5-b]xanthen-10-one	C16H12O6	-	433975.5867	188891.395
77	Quercetin-3-O-(6''-O-acetyl)glucoside	C23H22O13	-	922342.1633	387050.063
78	Tricin-7-O-saccharic acid	C23H22O14	-	355088.3607	158729.719
79	Kaempferol-3-O-(2'''-p-Coumaroyl)sophoroside-7-O-Glucoside	C42H46O23	-	38399.35667	16788.96033
80	6-Hydroxykaempferol-3,6-O-Diglucoside*	C27H30O17	-	2233126.83	635252.8333
81	Quercetin-3-O-rutinoside-7-O-rhamnoside	C33H40O20	-	395495.056	177975.6265
82	Avicularin(Quercetin-3-O-α-L-arabinofuranoside)*	C20H18O11	572-30-5	12073978.81	4846605.977
83	Luteolin-4'-O-glucoside	C21H20O11	6920-38-3	12281176.66	5890489.6
84	Chrysoeriol-7-O-rutinoside-5-O-glucoside	C34H42O20	-	63774.37844	20461.37967

Continued table S2: Major differential in common metabolites between L3 and L1 treatments under Mg regulation

Rank	Compounds	Formula	CAS	L3	L1
85	Persicoside	C23H26O11	28978-03-2	480015.6932	984408.0344
86	4'-O-Methyl-6-hydroxygallocatechin 3-O-(N-Ethylglutamine ester) 3'-Gallate	C30H32N2O14	-	268498.8083	753974.6783
87	Kaempferol 3-(3,6-Di-P-Coumaroylglucoside)*	C39H32O15	-	1246958.578	2745932.536
88	Chrysoeriol-7-O-(6''-feruloyl)glucoside	C32H30O14	-	24663.31183	67274.16494
89	3'-O-Methylgallocatechin 3-O-(N-Ethylglutamine ester) 4'-Gallate	C30H32N2O13	-	394186.6121	1140569.119
90	3'-O-Methyl-6-hydroxygallocatechin 3-O-(N-Ethylglutamine ester)	C23H28N2O10	-	1946577.588	4711544.621
91	Isoxanthopterin	C6H5N5O2	529-69-1	26623.34809	12535.52208
92	1,7-Dimethylxanthine	C7H8N4O2	611-59-6	4327560.596	1680200.353
93	Elaidolinolenic acid	C18H30O2	28290-79-1	6921013.998	1026348.041
94	2-Hydroxyphenol-1-O-glucosyl(6→1)rhamnoside	C18H26O11	-	107523.7987	9927.131333
95	1-O-Sinapoyl-β-D-glucose	C17H22O10	-	291943.1	117522.7727
96	Dihydroxybenzoyl xyloside	C12H14O8	-	939647.3184	26263.07633
97	Isomartynoside	C31H40O15	94410-22-7	13682703.68	4748238.297
98	Salicin 6'-Sulfate	C13H18O10S	-	13102164.93	141299.7203
99	3-(3-Hydroxyphenyl)-propionic acid	C9H10O3	621-54-5	1080475.978	470368.7882
100	Methyl caffeate	C10H10O4	3843-74-1	92255.33576	39467.19847
101	2,3-Dihydroxybenzoic Acid*	C7H6O4	303-38-8	1224227.598	405710.8588
102	2,5-Dihydroxybenzoic acid; Gentisic Acid*	C7H6O4	490-79-9	1224227.598	405710.8588
103	Methyl 3-(3-hydroxy-4-methoxyphenyl)propanoate	C11H14O4	129150-61-4	47457.2707	22405.104
104	O-Anisic acid (2-Methoxybenzoic acid)	C8H8O3	529-75-9	605746.0963	265868.0473

Continued table S2: Major differential in common metabolites between L3 and L1 treatments under Mg regulation

Rank	Compounds	Formula	CAS	L3	L1
105	Phenoxyacetic acid	C8H8O3	122-59-8	605746.0963	265868.0473
106	Salvianic acid B	C18H16O8	-	93330.66129	45432.02778
107	1,6-Di-O-galloyl-3-O-Feruloyl-β-D-glucose	C30H28O17	-	73863.94133	31712.69833
108	4-O-Glucosyl-sinapate	C17H22O10	-	70576.87111	35121.92733
109	Methyl gallate-4-O-β-D-glucoside	C14H18O10	-	3515239.122	1309906.244
110	Benzyl-(2''-O-glucosyl)glucoside*	C19H28O11	-	288788.5311	128464.2764
111	2-(3,4-Dihydroxyphenethoxy)-6-(3,4,5-trihydroxycaffeoyl)-O-arabinopyranosyl-D-glucose	C28H34O16	-	807935.8225	372178.7982
112	3-Dehydroshikimic Acid 5-(3-Methylsalicyloyl)Gallate	C22H18O11	-	4004893.732	9449670.668
113	3-Dehydroshikimic Acid 5-(3-Vanilloyl)Gallate	C22H18O12	-	5998940.552	14925542.59
114	Homovanillic alcohol; 4-Hydroxy-3-methoxyphenethanol	C9H12O3	2380-78-1	23459.12687	47872.79092
115	N-Methyl-Trans-4-Hydroxy-L-Proline	C6H11NO3	4252-82-8	2065075.332	1001625.798
116	γ-Glutamyltyrosine	C14H18N2O6	7432-23-7	596770.5117	186073.635

Table S3: Major differential in common metabolites between L4 and L1 treatments under Mg regulation

Rank	Compounds	Formula	CAS	L4	L1
1	2-Hydroxyphenol-1-O-glucosyl(6→1)rhamnoside	C18H26O11	-	55037.42167	9927.131333
2	9,16-Dihydroxypalmitic acid	C16H32O4	38076-49-2	2257799.109	1078530.253

Continued table S3: Major differential in common metabolites between L4 and L1 treatments under Mg regulation

Rank	Compounds	Formula	CAS	L4	L1
3	1-(2,3-dihydroxypropoxy)-3-(((2-(dimethylamino)ethoxy)(hydroxy) phosphoryl)oxy)propan-2-yl (E)-hexadec-9-enoate	C26H52NO9P	-	12723.42316	6225.849617
4	Monogalactosyldiacylglycerol	C45H70O10	-	101847.6714	29505.51287
5	Plumieride(Z)-P-Coumarate	C30H32O14	-	754392.4407	1570097.916
6	13-O-p-Coumaroylplumieride	C30H32O14	80416-52-0	821497.6599	2110374.105
7	N-Isobutyl-4,5-epoxy-2E-decaenamide	C14H25NO2	-	133683.3883	62480.48803
8	N-Isobutyl-2E,4E-dodedienamide	C16H29NO	-	40547.92505	13460.6074
9	Tri-p-coumaroyl Spermidine	C34H37N3O6	-	1097155.705	270258.1549
10	Candicine	C11H18NO+	6656-13-9	369511.932	831646.4466
11	2(3H)-Benzothiazolone	C7H5NOS	934-34-9	14392.45794	30563.79071
12	3,4-Dimethylellagic acid 4'-sulfate	C16H10O11S	-	208494.565	39460.26075
13	Gallic acid-1-O-xyloside	C12H14O9	-	2467435.233	35129.40523
14	Chebulanin	C27H24O19	166833-80-3	51831.14948	135995.259
15	L-Ascorbic acid (Vitamin C)	C6H8O6	50-81-7	15511333.17	363899.9985
16	D-Glucurono-6,3-lactone	C6H8O6	32449-92-6	719839.2653	222107.3857
17	2,5,8-trimethyldeca-2,4,7-triene-1,10-diol	C13H22O2	-	2058175.525	653802.6678
18	[(2R,3R,4S,5S)-4-hydroxy-5-(hydroxymethyl)-2-[[(2R,3S,4S,5R,6S)-3,4,5-trihydroxy-6-[2-[(1R)-4-methylcyclohex-3-en-1-yl]propan-2-yloxy]oxan-2-yl]methoxy]oxolan-3-yl]3,4,5-trihydroxybenzoate	C28H40O14	-	135292.0657	37180.40239
19	Delta-Tocopherol	C27H46O2	119-13-1	47521.24108	16413.45319
20	Menatetrenone (Vitamin K2)	C31H40O2	11032-49-8	1668399.926	4290680.977

Continued table S3: Major differential in common metabolites between L4 and L1 treatments under Mg regulation

Rank	Compounds	Formula	CAS	L4	L1
21	Prustomentoside C	C22H32O13	-	63243.61767	128443.4319
22	Medioresinol	C21H24O7	40957-99-1	99123.78271	25230.97944
23	Olivil Monoacetate	C22H26O8	1016974-78-9	47507.5319	100189.4522
24	1-Hydroxyanthraquinone	C14H8O3	129-43-1	139722.7996	54167.55567
25	Nobiletin (5,6,7,8,3',4'-Hexamethoxyflavone)	C21H22O8	478-01-3	1486946.677	509747.5945
26	Phloretin-4'-O-glucoside (Trilobatin)	C21H24O10	4192-90-9	2986724.839	1424808.792
27	Dihydrocharcone-4'-O-glucoside	C21H24O10	-	2924562.815	1415653.995
28	Dihydroxy-dimethoxyflavone	C17H14O6	-	109277.7183	23358.73337
29	3',4',5',5,7-Pentamethoxyflavone	C20H20O7	53350-26-8	204161.6261	36211.72542
30	5,7,8,4'-Tetramethoxyflavone	C19H18O6	6601-66-7	110499.341	32001.25953
31	3,5,7,3'4'-Pentamethoxyflavone	C20H20O7	1247-97-8	73187.83027	27810.557
32	Sinensetin (5,6,7,3',4'-pentamethoxyflavone)	C20H20O7	2306-27-6	80759.6612	34176.95056
33	Quercetin-3-O-rhamnoside(Quercitrin)	C21H20O11	522-12-3	6342703.406	2624302.903
34	Kaempferol 3-(2,4-Di-P-Coumaroylglucoside)*	C39H32O15	-	1093244.698	2780673.129
35	Quercetin-3-O-(6''-O-malonyl)glucoside	C24H22O15	-	4181.679533	13080.815
36	Kaempferol 3-(3,6-Di-P-Coumaroylglucoside)*	C39H32O15	-	1101846.423	2745932.536
37	Quercetin-3-O-(2''-O-galloyl)Arabinoside	C27H22O15	-	98318.33985	209764.4871
38	3'-O-Methylgallocatechin 3-O-(N-Ethylglutamine ester) 4'-Gallate	C30H32N2O13	-	475873.2861	1140569.119
39	2'-Deoxycytidine-5'-monophosphate	C9H14N3O7P	1032-65-1	170198.1536	51850.53362
40	Uric acid	C5H4N4O3	69-93-2	49649.61137	130383.9835
41	Dihydroxybenzoyl xyloside	C12H14O8	-	825453.7592	26263.07633

Continued table S3: Major differential in common metabolites between L4 and L1 treatments under Mg regulation

Rank	Compounds	Formula	CAS	L4	L1
42	Salicin 6'-Sulfate	C13H18O10S	-	11702348.77	141299.7203
43	Digalloylglucose*	C20H20O14	-	658116.8076	254351.4187
44	2,3-Dihydroxybenzoic Acid*	C7H6O4	303-38-8	912119.1803	405710.8588
45	2,5-Dihydroxybenzoic acid; Gentisic Acid*	C7H6O4	490-79-9	912119.1803	405710.8588
46	1,4-Di-O-galloyl-3-O-caffeoyl-β-D-glucose	C29H26O17	-	22302.33467	66666.53867
47	Homovanillic alcohol; 4-Hydroxy-3-methoxyphenethanol	C9H12O3	2380-78-1	22593.17453	47872.79092
48	γ-Glutamyltyrosine	C14H18N2O6	7432-23-7	821927.7762	186073.635
49	N-Acetylneuraminic acid	C11H19NO9	131-48-6	145741.3613	50910.02296
50	N-α-Acetyl-L-ornithine	C7H14N2O3	6205-08-9	278765.0112	583379.5534
51	Ile-Trp	C17H23N3O3	13589-06-5	167726.0737	450737.344

Table S4: Major differential in common metabolites between L5 and L1 treatments under Mg regulation

Rank	Compounds	Formula	CAS	L5	L1
1	Punicic acid (9Z,11E,13Z-octadecatrienoic acid)	C18H30O2	544-72-9	3434413	618432.2134
2	9S-Hydroperoxy-10E,12Z-octadecadienoic acid	C18H32O4	5502-91-0	439123	53955.81707
3	2-Aminooctadecane-1,16,18,18-tetraol	C18H39NO4	-	158541	70224.37546
4	2-Aminohexadecane-1,16,16-triol	C16H35NO3	-	1328839	364588.3942
5	Gingerglycolipid A	C33H56O14	145937-22-0	1714720	706054.7503

Continued table S4: Major differential in common metabolites between L5 and L1 treatments under Mg regulation

Rank	Compounds	Formula	CAS	L5	L1
6	2-Aminohexadecane-1,5,6-triol	C16H35NO3	-	177783	52298.23104
7	2-Aminododecane-1,3,4-triol	C12H27NO3	-	76814	23471.87
8	LysoPG 16:0	C22H45O9P	-	212489	63275.66804
9	Glucosyl 2,3-Dihydroxy-2-Methylbutanoic Acid	C11H20O9	-	3369872	943352.6649
10	kadsulactone A	C30H42O5	132296-71-0	725872	357216.8074
11	8(26),14(27)-onoceradiene-3β,21α-diol	C31H52O2	-	138674	47757.9752
12	2,3,19,23-Tetrahydroxyurs-12-en-28-oic acid	C30H48O6	-	3029122	865463.3234
13	2,19-Dihydroxy-3-oxours-12-en-28-oic acid	C30H46O5	176983-21-4	703478	302795.4592
14	Clerodendrin I	C33H44O14	-	352679	167409.5647
15	Bartsioside	C15H22O8	62133-72-6	2469082	1159725.683
16	2,3,19,23,24-Pentahydroxyolean-12-en-28-oic acid	C30H48O7	-	33984	8468.77145
17	1'S,4'S-4'-Dihydroabscisic acid-4'-O-β-malonylglucopyranoside	C24H34O12	-	160686	67543.017
18	Blumenol C glucoside	C19H32O7	135820-80-3	733684	211665.3785
19	Javanicolide C	C26H36O11	-	2471538	808941.8657
20	3-Hydroxyolean-12-ene-27,28-dioic acid (Cincholic acid)	C30H46O5	5948-32-3	66900	20782.64037
21	(6R,9S)-3-Oxo-α-ionol-β-D-glucopyranoside	C19H32O8	-	871959	423272.7984
22	Frehmaglutoside G	C21H36O9	-	219253	103959.4975
23	Geniposide	C17H24O10	24512-63-8	3946636	1531357.882
24	Genipin	C11H14O5	6902-77-8	318188	126212.2242
25	Dehydroxyampelopsisionoside	C19H32O7	-	634373	201367.2342
26	N-Feruloylputrescine N-glucoside	C20H30N2O8	-	738166	261224.4198

Continued table S4: Major differential in common metabolites between L5 and L1 treatments under Mg regulation

Rank	Compounds	Formula	CAS	L5	L1
27	Caffeoylagmatine	C14H20N4O3	-	1151069	570261.4873
28	Dendrocrepine*	C33H44N2O3	51020-39-4	42853	16499.0639
29	Sinapoylagmatine	C16H24N4O4	-	97050	45662.7765
30	Procyanidin A6	C31H28O12	-	1485760	626256.7277
31	Gallic acid-1-O-xyloside	C12H14O9	-	2870654	35129.40523
32	3-Ethyl-7-hydroxyphthalide	C10H10O3	-	1807856	723072.713
33	Roseoside	C19H30O8	54835-70-0	353250	152509.0787
34	L-Ascorbic acid (Vitamin C)	C6H8O6	50-81-7	52536796	363899.9985
35	D-Glucurono-6,3-lactone	C6H8O6	32449-92-6	1745065	222107.3857
36	2,5,8-trimethyldeca-2,4,7-triene-1,10-diol	C13H22O2	-	2569705	653802.6678
37	2-[3-[4-[1,3-dihydroxy-1-(4-hydroxy-3-methoxyphenyl)propan-2-yl]oxy-3-methoxy phenyl]propoxy]-6-(hydroxymethyl)oxane-3,4,5-triol	C26H36O12	-	4848155	1860691.883
38	Syringaldehyde; 4-Hydroxy-3,5-Dimethoxybenzaldehyde	C9H10O4	134-96-3	416189	167427.3233
39	[(2R,3R,4S,5S)-4-hydroxy-5-(hydroxymethyl)-2-[[(2R,3S,4S,5R,6S)-3,4,5-trihydroxy-6-[2-[(1R)-4-methylcyclohex-3-en-1-yl]propan-2-yloxy]oxan-2-yl] methoxy]oxolan-3-yl]3,4,5-trihydroxybenzoate	C28H40O14	-	212284	37180.40239
40	Manninotriose	C18H32O16	13382-86-0	34426	13783.77133
41	sanshodiol	C20H22O6	-	682990	268057.3396
42	Methoxy-erythro-Guaiacyl glycerol β-threo-syringyl glycerol ether	C22H30O11	-	449283	177899.9962
43	Secoisolariciresinol 4-O-glucoside	C26H36O11	-	2077881	634852.423
44	Erythro-Guaiacylglycerol-β-dihydroconiferyl Ether glucoside	C26H36O12	-	4848155	1860691.883

Continued table S4: Major differential in common metabolites between L5 and L1 treatments under Mg regulation

Rank	Compounds	Formula	CAS	L5	L1
45	glochidioboside	C26H34O11	-	1064716	341163.378
46	6-((4-(3-hydroxy-2-(4-(3-hydroxypropyl)-2-methylphenoxy)propoxy)-2-methoxyphenoxy)methyl)tetrahydro-2H-pyran-2,3,4,5-tetraol	C26H36O11	-	2348163	713634.1545
47	1-Hydroxypinoresinol-4'-O-Glucoside	C26H32O12	-	82661	30239.256
48	Matairesinol-4'-O-glucoside (Matairesinoside)	C26H32O11	23202-85-9	1429932	399987.5247
49	Sakuraresinol	C24H32O9	-	102069	41135.01333
50	2,6-di-O-galloylarbutin	C26H24O15	-	938736	434512.0567
51	(2S,3R,4S,5S,6R)-2-[4-[(1R,2R)-2-[4-[(3R,3aS,6R,6aS)-3-(4-hydroxy-3,5-dimethoxyphenyl)-1,3,3a,4,6,6a-hexahydrofuro[3,4-c]furan-6-yl]-2,6-dimethoxyphenoxy]-1,3-dihydroxypropyl]-2,6-dimethoxyphenoxy]-6-(hydroxymethyl)oxane-3,4,5-triol	C39H50O18	-	35786	16532.22925
52	Phloretin-2'-O-glucoside (Phlorizin)	C21H24O10	60-81-1	3598925	1563930.415
53	Diosmetin-7-O-rutinoside (Diosmin)	C28H32O15	520-27-4	6698475	3267272.27
54	Quercetin-3-O-glucuronide	C21H18O13	22688-79-5	518491	251998.3017
55	Phloretin-4'-O-glucoside (Trilobatin)	C21H24O10	4192-90-9	4164401	1424808.792
56	Epicatechin Tetramethyl Ether 3-Methylsulfate	C20H24O9S	-	8935510	4426756.911
57	Dihydrocharcone-4'-O-glucoside	C21H24O10	-	4062174	1415653.995
58	Robinetin	C15H10O7	490-31-3	7073152	3203887.373
59	Chrysoeriol-7-O-(6''-acetyl)glucoside	C24H24O12	-	443796	171190.3962
60	8,11-dimethoxy-2h-[1,3]dioxolo[4,5-b]xanthen-10-one	C16H12O6	-	550867	188891.395
61	Quercetin-3-O-rhamnoside(Quercitrin)	C21H20O11	522-12-3	6082531	2624302.903

Continued table S4: Major differential in common metabolites between L5 and L1 treatments under Mg regulation

Rank	Compounds	Formula	CAS	L5	L1
62	Morin-3-O-xyloside*	C20H18O11	-	12827013	5835769.653
63	5-Hydroxy-6,7,3',4',5'-Pentamethoxyflavone	C20H20O8	29215-55-2	13172	6466.029167
64	Kaempferol-3-O-(2'''-p-Coumaroyl)sophoroside-7-O-Glucoside	C42H46O23	-	49694	16788.96033
65	Avicularin(Quercetin-3-O-α-L-arabinofuranoside)*	C20H18O11	572-30-5	11848966	4846605.977
66	Tamarixetin-3-O-glucoside-7-O-rhamnoside	C28H32O16	-	7627113	2971716.515
67	2'-Hydoxy-5-methoxyGenistein-O-rhamnosyl-glucoside	C28H32O16	-	7627113	2971716.515
68	Sexangularetin-3-O-glucoside-7-O-rhamnoside	C28H32O16	-	7627113	2971716.515
69	Chrysoeriol-7-O-rutinoside-5-O-glucoside	C34H42O20	-	47170	20461.37967
70	1,7-Dimethylxanthine	C7H8N4O2	611-59-6	4338445	1680200.353
71	2'-Deoxycytidine-5'-monophosphate	C9H14N3O7P	1032-65-1	168630	51850.53362
72	Elaidolinolenic acid	C18H30O2	28290-79-1	10432838	1026348.041
73	2-Hydroxyphenol-1-O-glucosyl(6→1)rhamnoside	C18H26O11	-	102932	9927.131333
74	Protocatechuic acid glucosyl xyloside	C18H24O13	-	1476507	697158.8574
75	1,7-bis(4-hydroxy-3-methoxyphenyl)hept-1-ene-3-ol	C21H26O5	-	411706	204401.1686
76	1-O-Sinapoyl-β-D-glucose	C17H22O10	-	284584	117522.7727
77	Dihydroxybenzoyl xyloside	C12H14O8	-	931277	26263.07633
78	Isomartynoside	C31H40O15	94410-22-7	14167271	4748238.297
79	Salicin 6'-Sulfate	C13H18O10S	-	14805943	141299.7203
80	3-(3-Hydroxyphenyl)-propionic acid	C9H10O3	621-54-5	1125341	470368.7882
81	4-Hydroxyphenyl 6-O-(2-Methyl-3-Hydroxypropionyl)-Beta-D-Galactopyranoside	C16H22O9	-	532424	155014.3454

Continued table S4: Major differential in common metabolites between L5 and L1 treatments under Mg regulation

Rank	Compounds	Formula	CAS	L5	L1
82	Methyl caffeate	C10H10O4	3843-74-1	92927	39467.19847
83	Methyl 3-(3-hydroxy-4-methoxyphenyl)propanoate	C11H14O4	129150-61-4	52032	22405.104
84	Anthranilate-1-O-Sophoroside	C19H27NO12	-	497369	228769.098
85	1,6-Di-O-galloyl-2-O-Feruloyl- β -D-glucose	C30H28O17	-	117280	34394.579
86	2,6-Dihydroxy-4-isopropylphenyl-1-O- β -D-glucoside	C15H22O8	-	671811	326877.72
87	4-O-Glucosyl-sinapate	C17H22O10	-	70787	35121.92733
88	2-(3,4-Dihydroxyphenethoxy)-6-(3,4,5-trihydroxycaffeoyl)-O-arabinopyranosyl-D-glucose	C28H34O16	-	833190	372178.7982
89	Syringin	C17H24O9	118-34-3	586983	288045.7652
90	N-Acetylneuraminic acid	C11H19NO9	131-48-6	141613	50910.02296
91	9,16-Dihydroxypalmitic acid	C16H32O4	38076-49-2	500721	1078530.253
92	1-(9Z-Octadecenoyl)-2-(9-oxo-nonanoyl)-sn-glycero-3-phosphocholine	C35H66NO9P	-	208830	455746.618
93	LysoPE 18:2	C23H44NO7P	-	1927239	4336513.795
94	1- α -Linolenoyl-glycerol*	C21H36O4	-	913873	2206562.042
95	LysoPE 16:1*	C21H42NO7P	-	45837	95229.14879
96	LysoPC 19:0	C27H56NO7P	108273-88-7	41195	96617.12526
97	Coixenolide	C38H70O4	-	26693	73034.35308
98	LysoPE 18:0(2n isomer)	C23H48NO7P	-	23522	51806.2009
99	PI(18:1/0:0)	C27H51O12P	-	174855	553537.9682
100	Jasmonic acid	C12H18O3	77026-92-7	173226	591098.167

Continued table S4: Major differential in common metabolites between L5 and L1 treatments under Mg regulation

Rank	Compounds	Formula	CAS	L5	L1
101	Ent-3 β -Acetoxyisopimar-15-8 β -ol	C22H36O3	-	1083565	3293585.532
102	Viteagnusin B	C22H36O3	-	1083565	3293585.532
103	Lupa-1,20(29)-dien-3-one (Glochidone)	C30H46O	-	249339	903093.9687
104	14-hydroxy-14-(hydroxymethyl)-5,9-dimethyltetracyclo[11.2.1.01,10.04,9] hexadecane-5-carboxylic acid	C20H32O4	-	308196	625476.2701
105	Ajugalaevigatic acid	C22H36O4	-	4066551	9283382.317
106	(E)-Cinnamamide	C9H9NO	22031-64-7	68611	138610.2114
107	Chebulanin	C27H24O19	166833-80-3	31367	135995.259
108	3,7-Dimethyl-2,6-Octadienyl 6-O-(beta-D-Xylopyranosyl)-beta-D-glucopyranoside	C21H36O10	-	3458365	6929941.121
109	3,4-methylenedioxy cinnamyl alcohol	C10H10O3	58095-76-4	888281	2078488.488
110	9,12-Octadecadienoic acid,ethyl ester	C20H36O2	6114-21-2	122807	294111.0812
111	Inositol*	C6H12O6	87-89-8	2334694	5067723.083
112	2,7-Dimethyl-2,4-Octadiene-1,8-Diol 8-O-B-D-Glucopyranoside	C16H28O7	-	270266	572427.1071
113	2,7-Dimethyl-2,4-Octadiene-1,8-Diol 8-O-B-D-(6''-Malonylglucoside)	C19H30O10	-	263577	527881.9374
114	Dehydrodiconiferyl alcohol	C20H22O6	4263-87-0	276240	622513.0667
115	Schisantherin J	C35H40O11	135432-28-9	1265611	4641422.593
116	Apigenin-6,8-di-C-glucoside-4'-O-glucoside	C33H40O20	-	165233	343725.207
117	Persicoside	C23H26O11	28978-03-2	434851	984408.0344
118	4'-O-Methyl-6-hydroxygallocatechin 3-O-(N-Ethylglutamine ester) 3'-Gallate	C30H32N2O14	-	290591	753974.6783
119	Castanoside B[Kaempferol-3-O-(4'',6''-di-p-coumaroyl)mannoside]	C39H32O15	-	563766	1539942.058
120	Epigallocatechin 3-O-Cinnamate	C24H20O8	-	1964308	4052406.529

Continued table S4: Major differential in common metabolites between L5 and L1 treatments under Mg regulation

Rank	Compounds	Formula	CAS	L5	L1
121	Methylhesperidin	$C_{29}H_{36}O_{15}$	11013-97-1	871806	1760189.271
122	Kaempferol 3-(3,6-Di-P-Coumaroylglucoside)*	$C_{39}H_{32}O_{15}$	-	1204998	2745932.536
123	3'-O-Methyl-6-hydroxygallocatechin 3-O-(N-Ethylglutamine ester) 3'-Gallate	$C_{30}H_{32}N_2O_{14}$	-	966585	2407557.842
124	3'-O-Methylgallocatechin 3-O-(N-Ethylglutamine ester) 4'-Gallate	$C_{30}H_{32}N_2O_{13}$	-	323899	1140569.119
125	3'-O-Methyl-6-hydroxygallocatechin 3-O-(N-Ethylglutamine ester)	$C_{23}H_{28}N_2O_{10}$	-	2169268	4711544.621
126	Uric acid	$C_5H_4N_4O_3$	69-93-2	60021	130383.9835
127	Benzyl salicylate	$C_{14}H_{12}O_3$	118-58-1	300412	641571.3595
128	3-O-Methylgallic Acid	$C_8H_8O_5$	3934-84-7	50325222	115101910.2
129	3-O-p-Coumaroylshikimic acid	$C_{16}H_{16}O_7$	-	69164	180496.6345
130	1,4-Di-O-galloyl-3-O-caffeoyl-β-D-glucose	$C_{29}H_{26}O_{17}$	-	15271	66666.53867
131	1,2-Di-O-galloyl-5-O-Cinnamoyl-β-D-glucose	$C_{29}H_{26}O_{15}$	-	53635	136270.1279
132	(S)-4-amino-5-(butylamino)-5-oxopentanoic acid	$C_9H_{18}N_2O_3$	-	398274	817220.7334
133	Jasmonoyl-L-Isoleucine	$C_{18}H_{29}NO_4$	120330-93-0	137474	297581.8747
134	γ-Glutamyl-L-valine	$C_{10}H_{18}N_2O_5$	-	78798	225767.3115

Table S5: Expression of genes related to EGCG biosynthesis metabolism in tea tree after treatment with different concentrations of magnesium

Gene name	Gene id	Treated magnesium concentration（mmol/L）				
		0	0.15	0.45	0.6	0.9
PAL	HD.11G0022880	0	0	0.04	0.046667	0
PAL	HD.01G0005520	70.76	79.74	94.43	77.26	75.99
PAL	HD.02G0024350	48.52	52.74	42.18	48.44	50.02
PAL	HD.14G0008830	8.65	9.57	10.78	10.4	11.7
PAL	HD.02G0024340	4.633	6.32	4.33	4.65	4.92
PAL	HD.13G0009900	351.14	336.91	338.33	310.88	353.98
PAL	HD.06G0008610	261.57	247.15	283.36	234.72	263.03
C4H	HD.06G0017130	142.46	124.8	171.28	159.89	142.30
4CL	HD.09G0016320	0.94	0.86	0.75	0.94	0.94
4CL	HD.1504232	11.34	9.88	10.55	9.70	8.98
4CL	HD.04G0016430	0.07	0.15	0.07	0.07	0.16
4CL	HD.15G0010720	0.07	0	0.02	0.08	0.03
4CL	HD.15G0008250	66.38	67.68	74.12	62.75	60.54
4CL	HD.02G0027390	151.85	157.3	174.14	184.76	199.17
4CL	HD.13G0010220	47.92	48.03	52.94	45.7	46.02
4CL	HD.09G0013420	0.69	1.11	2.4	2.01	1.83
4CL	HD.04G0003270	68.41	73.57	76.41	74.84	72.98
CHS	HD.10G0022640	1340.51	1282.58	1517.24	1365.50	1480.00
CHS	HD.04G0020800	0.04	0.08	0.08	0.01	0.01

Continued table S5: Expression of genes related to EGCG biosynthesis metabolism in tea tree after treatment with different concentrations of magnesium

Gene name	Gene id	Treated magnesium concentration（mmol/L）				
		0	0.15	0.45	0.6	0.9
CHI	HD.02G0022260	173.89	158.34	200.50	172.97	168.69
CHI	HD.01G0011100	128.98	130.09	166.26	148.52	154.25
F3H	HD.01G0028710	12.22	7.69	16.91	11.27	10.70
F3H	HD.12G0008190	0.34	0.2	0.17	0.44	0.52
F3H	HD.10G0001940	538.70	515.98	575.02	558.30	574.51
DFR	HD.06G0036230	17.88	16.52	19.24	14.35	21.79
DFR	HD.04G0026220	233.73	199.87	272.04	198.61	176.01
DFR	HD.06G0036240	2.44	1.45	2.25	2.31	1.12
DFR	HD.06G0036220	1.03	1.29	1.04	0.99	1.08
ANS	HD.09G0021220	2.95	2.64	3.94	4.13	3.68
ANS	HD.09G0021230	9.48	10.80	23.45	15.57	10.94
ANS	HD.12G0014070	60.30	49.53	96.78	54.44	47.67
ANR	HD.07G0023630	921.47	941.51	977.31	1022.14	1006.94
ANR	HD.12G0016700	368.87	358.7	314.72	272.45	281.98
F3'H	HD.15G0015490	566.85	550.09	700.24	559.92	738.13
F3'5'H	HD.13G0004300	14.89	12.83	16.42	13.13	15.18
aroB	HD.01G0028400	61.58	61.60	63.30	60.95	64.83
aroDE	HD.03G0015950	0.08	0.17	0.08	0.08	0.13
aroDE	HD.09001409	1.52	1.62	3.03	2.22	1.95
aroDE	HD.03G0002480	3.21	3.32	3.44	2.94	2.83

Continued table S5: Expression of genes related to EGCG biosynthesis metabolism in tea tree after treatment with different concentrations of magnesium

Gene name	Gene id	Treated magnesium concentration（mmol/L）				
		0	0.15	0.45	0.6	0.9
SCPL	HD.05G0027520	4.41	3.14	5.74	3.56	2.91
SCPL	HD.01G0041070	38.5	39.82	37.06	30.88	37.39
SCPL	HD.1307844	17.95	10.78	20.32	9.73	7.59
SCPL	HD.03001711	15.93	11.26	19.93	10.59	9.17
